# Does “Dr. Google” improve discussion and decisions in small animal practice? Dog and cat owners use of internet resources to find medical information about their pets in three European countries

**DOI:** 10.3389/fvets.2024.1417927

**Published:** 2024-06-19

**Authors:** Svenja Springer, Thomas Bøker Lund, Sandra A. Corr, Peter Sandøe

**Affiliations:** ^1^Messerli Research Institute, Department of Interdisciplinary Life Sciences, University of Veterinary Medicine, Vienna, Austria; ^2^Department of Food and Resource Economics, University of Copenhagen, Frederiksberg, Denmark; ^3^Division of Small Animal Clinical Sciences, School of Biodiversity, One Health and Veterinary Medicine, University of Glasgow, Glasgow, United Kingdom; ^4^Department of Veterinary and Animal Sciences, University of Copenhagen, Frederiksberg, Denmark

**Keywords:** internet, pet health information, “Google”, veterinary advice, small animal practice, dog owners, cat owners, representative questionnaire-based survey

## Abstract

Modern dog and cat owners increasingly use internet resources to obtain information on pet health issues. While access to online information can improve owners’ knowledge of patient care and inform conversations with their veterinarian during consultations, there is also a risk that owners will misinterpret online information or gain a false impression of current standards in veterinary medicine. This in turn can cause problems or tensions, for example if the owner delays consulting their veterinarian about necessary treatment, or questions the veterinarian’s medical advice. Based on an online questionnaire aimed at dog and cat owners in Austria, Denmark and the United Kingdom (*N* = 2117) we investigated the use of internet resources to find veterinary medical information, the type of internet resources that were used, and whether owner beliefs explain how often they used the internet to find medical information about their pet. Approximately one in three owners reported that they never used internet resources prior to (31.7%) or after (37.0%) a consultation with their veterinarian. However, when owners do make use of the internet, our results show that they were more likely to use it before than after the consultation. The most common internet resources used by owners were practice websites (35.0%), veterinary association websites (24.0%), or ‘other’ websites providing veterinary information (55.2%). Owners who believe that the use of internet resources enables them to have a more informed discussion with their veterinarians more often use internet resources prior to a consultation, whereas owners who believed that internet resources help them to make the right decision for their animal more often use internet resources after a consultation. The results suggest that veterinarians should actively ask pet owners if they use internet resources, and what resources they use, in order to facilitate open discussion about information obtained from the internet. Given that more than a third of pet owners use practice websites, the findings also suggest that veterinarians should actively curate their own websites where they can post information that they consider accurate and trustworthy.

## 1 Introduction

The development of the internet has had an enormous impact on the ease of access to information on most aspects of life. This is also the case when it comes to owners’ care of their companion animals. According to studies, dog and cat owners increasingly acquire information about pet health issues from various internet resources including veterinary medical websites, social media or blogs. For example, in a study among dog owners in the United Kingdom from 2013, Kuhl et al. (1) investigated the types of information sources dog owners use to research all aspects of their dog(s) care. Their results show that 71% of all surveyed dog owners indicate that they conduct a general internet search to find out about different aspects of their dog(s) care, compared to 66% of owners who sought such information from their veterinary surgeon (1). Similarly, results of a study of pet owners in the United Kingdom showed that owners were more likely to make use of the internet (79%) than to contact their veterinarian (72%) when looking for pet health information (2). These findings are also reflected in a transnational study of small animal veterinarians in Austria, Denmark and the United Kingdom, where approximately half of the veterinarians estimated that many of their clients use internet resources to find veterinary medical information (3).

The literature highlights both risks and benefits of clients using the internet to obtain information. While the improvement of clients’ knowledge and understanding of patient care and their increased acceptance of advanced diagnostics and treatments are seen as positive developments, increased expectations, misleading impressions of standards of care or misinterpretation of online information are highlighted as potentially negative aspects (3–7). In addition, studies show that veterinarians are increasingly experiencing situations where clients question their medical advice based on information obtained from the internet (3, 4, 6). This, in turn, can have an impact not only on veterinary decision-making processes, but also on the relationship between the client and the veterinarian.

In light of this, the aim of the present study was to gain insights into cat and dog owners’ beliefs about, and use of, internet resources to find veterinary medical information (from here on medical information) about their pet. Further, it was our aim to investigate how often owners use the internet prior to vs. after a veterinary consultation, as the timing is likely to be influenced by the purpose – for example, owners may consult the internet prior to a consultation to facilitate a more informed discussion with their veterinarian or even challenge their veterinarian to justify medical recommendations. On the other hand, they might consult the internet after a consultation to help them to decide what is right for their animal in light of what the veterinarian recommended, or to buy medications or services at a lower price than that offered by the veterinarian.

Although studies on the use of internet resources already exist, the present study goes beyond previous research in the following six ways: (i) The study includes three different European countries, Austria, Denmark, and the United Kingdom, that differ in two important dimensions that may impact the use of internet resources in the veterinary context: First, the countries differ with respect to citizens’ daily use of the internet: in 2020, in the United Kingdom and Denmark, 94% of the population used the internet on a daily basis (8, 9), in contrast to only 75% of the population in Austria (10). (ii) Second, the populations studied differ with respect to the level of general trust. For instance, data of the European Quality of Life Survey measured the general level of trust using data from the question “Would you say that most people can be trusted?” Results reveal that Danish citizens have a higher level of general trust (score of 6.97) compared to Austrian and United Kingdom citizens (score of 5.25 and 5.47, respectively) (11). These differences may be reflected in the number of clients in each country who use the internet to check medical information, or question veterinarians’ professional advice. (iii) While previous studies have been based on convenience samples of animal owners, the present study is based on samples that are fairly representative for key demographics of owners such as gender, age, and regional areas in all countries studied. (iv) The samples includes both dog and cat owners, which allows us to investigate whether the animal species (dog or cat) has an impact on whether and how often clients use internet resources prior to or after a consultation with their veterinarian. (v) While previous research has focused on the identification of possible differences among pet owners’ use of internet resources with respect to their age, gender, or education level (2, 7), the present study takes into account additional important aspects such as clients’ emotional attachment to their pet, their living situation (living alone or not), and whether they work in the veterinary sector. These factors have not previously been considered, but it seems reasonable to expect that these factors may impact clients’ use of internet resources to find medical information. For example, a strong emotional bond may increase the likelihood that an owner would use the internet to search for medical information to improve their pet’s care. (vi) The design of the present study is based on prior research conducted with small animal veterinarians in Austria, Denmark and the United Kingdom (3), enabling comparison between the results of the two studies to explore the differing perspectives on the use of internet resources to obtain medical information.

Therefore, we formulated the following six research questions: (1) How often do dog and cat owners use internet resources to find medical information *prior* to or *after* a consultation with the veterinarian? (2) What type of internet resources do they use to search for medical information? (3) How many owners have disagreed with the veterinarian’s professional advice based on information they had obtained from the internet? (4) What are owners’ beliefs about the use of internet resources to find medical information? (5) To what extent do owner characteristics, including sociodemographic features and emotional attachment to their pet explain *whether* they use internet resources? (6) And, to what extent do the aforementioned owner characteristics and beliefs about the use of internet resources explain *how often* they use internet resources prior to or after a consultation? Throughout, we highlight the main similarities and differences between owners in the three countries.

## 2 Materials and methods

### 2.1 Target population and recruitment of participants

The present study forms a part of a larger body of work on different aspects in modern small animal practice including, e.g., investigations of how dog and cat owners view advanced veterinary care (12) and telemedicine (13) in small animal practice. Participants were recruited by NORSTAT, a company that manages citizen panels in several European countries where the members of these panels have agreed to receive invitations to participate in research. The invitees were recruited from NORSTAT’s pre-established citizen panels in the three study countries, through a random draw of panel members. In Denmark, panel members were recruited through a mixture of telephone interviews (random digit dialling), and online sources (typically internet pages). In the United Kingdom and in Austria, panel members were only recruited through online sources. During data collection, NORSTAT continuously monitored the number of respondents that had completed the questionnaire across different age groups, geographical locations, and gender. If particular groups were under-represented (compared with the countries’ census), extra invitations were issued to this group (still using the random draw principle within the group). The aim was to gather samples that were as representative as possible of the general population of Austrian, Danish and United Kingdom citizens. Data were collected from 11th to 25th of March 2022 in Austria, from 11th to 24th of March 2022 in Denmark and from 8th to 23rd of March 2022 in the United Kingdom. In total, 17,747 citizens were invited to participate in the study: 5,207 in Austria, 6,075 in Denmark and 6,465 in the United Kingdom. The invitation provided information about the background of the study, the participating universities, ethical approval, estimated time for questionnaire completion and participant’s rights during the reply process. The entire project was approved by The Research Ethics Committee of SCIENCE and HEALTH at the University of Copenhagen (ReF: 504–010300/22–5000). We ensured informed consent by explaining the purpose of the study on the opening page and informing participants that by clicking on the button “Next,” they confirmed that they were over 17 years old, and consented to participate in the survey.

### 2.2 Eligible participants for this study

A total of 4,885 individuals clicked on the survey link, but 275 responses were subsequently removed because they failed to complete the questionnaire (dropout rate 5.62%). This gave a final sample size of 4,610 (1,500 Austrians, 1,552 Danes and 1,558 United Kingdom citizens.) The response rate was 30.3% for Austria, 27.5% for Denmark and 25.3% for United Kingdom. Since the target group of the present study was people owning dog(s) and/or cat(s) at the time of the survey, and not all respondents had pets, only a sub-set of the sample was used in this study. The final sample size used in the present study comprised 2,117 owners of dogs and/or cats (Austria: *n* = 800; Denmark: *n* = 626; United Kingdom: *n* = 691). Detailed information about the socio-demographic factors and the number of dog and/or cat owners per country is listed in Supplementary Table 1.

### 2.3 Survey development

Items related to dog and cat owners’ use of internet resources to obtain medical information were developed as a follow up to a transnational questionnaire study revealing veterinarians’ attitudes toward clients’ use of internet resources to obtain medical information (3). In addition, a literature review was conducted that served as a further basis for the development of items (2, 4, 6, 7). The questionnaire was developed in English, and a two-step translation and back-translation procedure was then used to produce versions to be used in Austria and Denmark. In a first step, the English questionnaire was translated into German and Danish. In a second step, the translated German and Danish versions were translated back into English by a second bilingual person. Subsequently, the retranslations were compared with the original English version, and in the case of mismatches, alterations were made in the translated versions in consultation with translators.

In addition, the questionnaire underwent two stages of cognitive pre-testing to identify whether the content led to uncertainties or misunderstandings by the respondents. In the first stage, 15 cognitive interviews (14, 15) were conducted with five owners in each country who varied with respect to their age, gender and whether they owned dog(s), cat(s) or both. In a second stage, an online pre-test phase was conducted with 123 Austrian citizens (including 34 dog and 39 cat owners), 152 Danish citizens (including 30 dog and 24 cat owners) and 117 United Kingdom citizens (including 41 dog and 40 cat owners). All relevant comments that were likely to improve the quality of data were considered and incorporated into the final version of the questionnaire in all three languages.

### 2.4 Survey measurements

The questionnaire consisted of three sections (Supplementary Data Sheet 1). In the following description, only questions and items of relevance to the present paper are considered.

The first section, A, included 19 closed-ended sociodemographic questions as well as questions related to the owned dog(s) and/or cat(s). A main explanatory variable for the present study is owners’ emotional attachment to their animals which we measured using the Lexington Attachment to Pets Scale (LAPS) (16, 17). The LAPS includes 23 statements such as “I believe my pet is my best friend” or “I think my pet is just a pet.” Respondents could answer the statements using a 4-point Likert scale ranging from 1 “Strongly disagree” to 4 “Strongly agree.” In case respondents had both a cat and a dog, they were asked which species their favorite pet was, and instructed to think about that pet when responding to the 23 LAPS statements. We used the information about their favorite pet to construct a further main explanatory variable, namely animal species (i.e., whether the owner has a cat or a dog). In total, 18.6% of pet owners reported having both dog(s) and cat(s) (see Supplementary Table 1). If the respondents who had both dog(s) and cat(s) identified the cat as being their favorite pet, they were ascribed to be cat owners, and vice versa if their favorite was a dog. Since some of the owners did not choose a cat or a dog as their favorite animal, but for example a horse, these owners were excluded and the eligible sample size decreased slightly by 31 respondents (1.5%).

The second section, B, covered emerging topics in modern small animal practice, including the use of internet resources to find medical information. The first two questions in this section asked owners how often they use internet resources prior to and after a consultation with their veterinarian. Answer options were “Never,” “Occasionally,” “Frequently,” “Always” and “I do not know.” If respondents chose “Occasionally,” “Frequently,” “Always” and “I do not know,” they were subsequently asked which resources they have used. Seven options were provided: “Blogs and chat rooms,” “Social media,” “Websites providing medical information,” “University websites,” “Practice website,” “Veterinary association websites” and ‘Other,” and respondents could tick all that applied. Further, seven statements were presented to explore owners’ beliefs about the use of internet resources to find medical information. Respondents were able to indicate their level of agreement with each statement through one of eight answer options from 1 “Strongly disagree” up to 7 “Strongly agree” and 8 “I do not know.” The final question of the sub-section asked whether respondents ever disagreed with the veterinarians’ professional advice based on information obtained from the internet - answer options were “Yes,” “No” and “I do not know”.

### 2.5 Data analysis

IBM® SPSS® Statistics version 29.0 (IBM® SPSS® Statistics, Chicago, IL, United States) was used for all analyses. Univariate descriptive statistics were presented in tables or text. For bivariate analysis, Kruskal-Wallis *H* Test were conducted to test whether the frequency distribution differed between Austria, Denmark and the United Kingdom with respect to owners’ use of internet resources to find medical information prior to or after a consultation with the veterinarian. Chi-Square Tests were performed to test whether owners’ use of different types of internet resources differed between the three countries. Since the data for the seven statements reflecting owners’ beliefs about the use of internet resources were not normally distributed, Kruskal-Wallis *H* tests were performed to test whether the frequency distribution differed between the three countries. Related-Samples Wilcoxon Signed Ranks Test were conducted for each country to test whether differences could be identified between the use of internet resources prior to versus after a consultation. Reporting of proportions, means and standard deviations were conducted with weighted data to mitigate the effects of any sample imbalances and to bring them more in line with the number of dog and cat owners in the three countries. We considered *p*-values below 0.05 as statistically significant. Bonferroni correction was employed for multiple comparison. Detailed explanations can be found in the notes to the tables where Bonferroni correction was employed.

To examine the effect of animal- and owner-related aspects on whether owners used internet resources to find medical information, binary regression analysis were conducted for each country. In doing so, we combined the two variables [use of internet resources both (1) prior to and (2) after a consultation] to identify owners who never used internet resources prior to or after a consultation. We then created a binary variable for the regression 0 = no use of the internet prior to and after a consultation; 1 = use of the internet (including the answer options: occasionally/frequently/always/I do not know). The following explanatory variables were inserted as continuous predictors in the regressions: age and emotional attachment to the animal (LAPS). Gender of the owner (1 = male, 2 = female), animal species (1 = dog, 2 = cat), working in the veterinary field (1 = yes, 2 = no) and living alone (1 = yes, 2 = no) were inserted as categorical variables.

To investigate to what extent owner characteristics and beliefs about the use of internet resources explain *how often* they use internet resources prior to or after a consultation, both variables, (1) use prior to, and (2) after a consultation, respectively – were of importance. For each country, we therefore ran two ordinal regressions where the two variables were inserted as dependent variables. Owners’ socio-demographic characteristics and animal-related factors were inserted as predictor variables (i.e., the same predictor variables described in the former paragraph). Two additional statements that reveal clients’ beliefs about the use of the internet were included as predictor variables in the regression analysis regarding use of internet resources to find medical information *prior* to a consultation: “The use of internet resources (a) enables me to have a more informed discussion with my vet” and (b) “enables me to challenge my vet to justify their recommendations.” We only included two of possible seven items to avoid inclusion of highly correlated statements, and made the decision about item inclusion based on correlation analyses conducted prior to the regression analysis. For the regression analysis related to the use of internet resources to find medical information *after* a consultation, we ran similar tests to detect the most important items and included the items: “The use of internet resources (a) helps me to make the right decision for my animal” and (b) “enables me to buy some medication(s) more cheaply (e.g., flea treatments, wormers)”.

## 3 Results

### 3.1 Frequency of owners’ use of internet resources to find medical information prior to or after a consultation with the veterinarian

Although approximately one third of owners reported that they never use internet resources prior to (31.1%) or after (37.0%) a consultation, those owners who do use them were significantly more likely to do so prior to than after a consultation with their veterinarian (*p* < 0.001) (Table 1). However, for the United Kingdom, there was no significant difference in the frequency of owners’ use of internet resources prior to or after a consultation (*p* = 0.394). Comparison between countries show that Danish owners make significantly less use of internet resources after the veterinary consultation compared to Austrian (*p* < 0.001) and United Kingdom owners (p < 0.001).

**Table 1:** Frequency of owners’ use of internet resources to find medical information prior to or after a consultation with the veterinarian.

	All countries (*N* = 2117)	Austria (*n* = 800)	Denmark (*n* = 626)	United Kingdom (*n* = 691)	Test^*^ (Differences between countries for frequency of use of internet resources prior and after consultation)
Prior to a consultation					
Never	649 (31.1)	214 (26.8)	220 (35.3)	215 (30.2)	H(2) = 5.363, *p* = 0.068
Occasionally	855 (39.7)	382 (47.3)	219 (34.6)	254 (35.8)	
Frequently	316 (14.7)	122 (14.1)	87 (14.1)	107 (16.1)	
Always	166 (7.9)	50 (5.9)	50 (7.9)	66 (10.2)	
I do not know	131 (6.5)	32 (4.3)	50 (8.1)	49 (7.7)	
After a consultation					
Never	784 (37.0)	251 (32.9)	298 (47.4)	235 (32.4)	H(2) = 58.898, *p***< 0.001**^a^ AT vs. DK: *p***< 0.001** AT vs. UK:*p *= < 0.810 DK vs. UK: *p***< 0.001**
Occasionally	756 (35.9)	349 (43.9)	178 (28.8)	229 (33.2)	
Frequently	288 (13.3)	127 (14.4)	54 (8.6)	107 (16.2)	
Always	132 (6.2)	40 (4.4)	26 (4.0)	66 (10.0)	
I do not know	157 (7.7)	33 (4.3)	70 (11.2)	54 (8.3)	
Test^**^ (Differences between after and prior regarding the frequency of consultation for each country)	*Z* = -6.250, *p*** < 0.001**	*Z* = -2.245, *p*** < 0.025**	*Z* = -7.969, *p*** < 0.001**	*Z* = -0.852, *p* = 0.394	

Number of respondents (N,n) and analyses with inferential statistics were calculated with unweighted data. Proportions were calculated with weighted data; rounding errors lead to some differences between rounded-off numerical values and actual values. *Kruskal-Wallis H test (answer option “I do not know” was excluded from these analyses). **Related-Samples Wilcoxon Signed Ranks Test (answer option “I do not know” was excluded from these analyses). Significant p-values are highlighted in bold.

### 3.2 Type of internet resources used to search for medical information

Owners who indicated that they use internet resources were subsequently asked what type of internet resources they use. Over half of the surveyed owners (55.2%) make use of websites providing medical information, 35% use practice websites, and 24% use veterinary association websites (Table 2). Considering differences between countries, our results show that Danish owners significantly more often use websites providing medical information (p_AT/UK_ < 0.001) and practice websites (p_AT_ = 0.003, p_UK_ < 0.001) compared to Austrian and United Kingdom owners. United Kingdom owners significantly more often make use of social media platforms compared to Austrian and Danish owners (p_AT/DK_ < 0.001).

**Table 2:** Internet resources used to find veterinary medical information.

Nr.		All countries (*N* = 1606)	Austria (*n* = 657)	Denmark (*n* = 432)	United Kingdom (*n* = 517)	Test^*^
1	Websites providing veterinary medical information	894 (55.2)	345 (52.1)	278 (64.1)	271 (51.9)	*χ*^2^(2) = 18.066, ***p* < 0.001**^a^ AT vs. DK: *χ*^2^(1) = 14.926, ***p* < 0.001** AT vs. UK: *χ*^2^(1) = 0.001, *p* = 0.975 UK vs. DK: *χ*^2^(1) = 13.746, ***p* < 0.001**
2	Practice website	570 (35.0)	222 (32.8)	188 (42.3)	160 (31.9)	*χ*^2^ (2) = 17.650, ***p* < 0.001**^a^ AT vs. DK: *χ*^2^(1) = 10.508, ***p* = 0.001** AT vs. UK: *χ*^2^(1) = 1.065, *p* = 0.302 UK vs. DK: *χ*^2^(1) = 16.014, ***p* < 0.001**
3	Veterinary association websites	391 (24.0)	150 (21.7)	101 (23.4)	140 (27.2)	*χ*^2^(2) = 3.135, *p* = 0.209
4	Social media (e.g., Facebook, Twitter)	295 (18.5)	112 (16.4)	52 (12.4)	131 (25.7)	*χ*^2^(2) = 29.065, ***p* < 0.001**^a^ AT vs. DK: *χ*^2^(1) = 5.114, ***p* = 0.024** AT vs. UK: *χ*^2^(1) = 12.118, ***p* < 0.001** UK vs. DK: *χ*^2^(1) = 26.752, ***p* < 0.001**
5	Blogs and chat rooms	195 (11.7)	102 (14.4)	31 (7.4)	62 (11.9)	*χ*^2^(2) = 17.047, ***p* < 0.001**^a^ AT vs. DK: *χ*^2^(1) = 16.946, ***p* < 0.001** AT vs. UK: *χ*^2^(1) = 3.005, *p* = 0.083 UK vs. DK: *χ*^2^(1) = 6.176, ***p* = 0.013**
6	University website	146 (8.8)	81 (11.5)	33 (7.4)	32 (6.6)	*χ*2 (2) = 14.702, ***p* < 0.001**^a^ AT vs. DK: *χ*2(1) = 6.116, ***p* = 0.013** AT vs. UK: *χ*2(1) = 12.536, ***p* < 0.001** UK vs. DK: *χ*2(1) = 0.775, *p* = 0.379
7	Other	216 (13.6)	93 (14.1)	71 (17.0)	52 (10.3)	*χ*2 (2) = 8.698, ***p* < 0.013**^a^

Number of respondents (N,n) and analyses with inferential statistics were calculated with unweighted data. Proportions were calculated with weighted data; rounding errors lead to some differences between rounded-off numerical values and actual values. *Pearson Chi-Square Test. ^a^Bonferroni correction was applied for significant results; as there are seven tests being made one for each type of internet resource used to find medical information, alpha was divided by 7 (*n* = 7): 0.05/7 = 0.007, i.e., each test is tested against a level of 0.007. Significant p-values are highlighted in bold.

### 3.3 Challenging the veterinarian’s advice

Another aim of the study was to identify whether owners have ever disagreed with a veterinarian’s professional advice based on information they had obtained from the internet. Overall, only a minority of owners (12.1%) indicated that they disagreed with their veterinarian’s advice (Table 3). By country, the proportion of Danish owners who reported such disagreement (4.9%) was significantly lower than the proportion of owners in Austria (13.6%, *p* < 0.001) or the United Kingdom (16.1%, p < 0.001).

**Table 3:** Information from owners on whether they have ever disagreed with the veterinarian’s professional advice based on information from the internet.

	All countries (*N* = 1606)	Austria (*n* = 657)	Denmark (*n* = 432)	UK (*n* = 517)	Test^*^
Yes	196 (12.1)	96 (13.6)	22 (4.9)	78 (16.1)	*χ*^2^(2) = 28.045, ***p* < 0.001** AT vs. DK: *χ*^2^(1) = 28.863, ***p* < 0.001** AT vs. UK: *χ*^2^(1) = 0.030, *p* = 0.862 UK vs. DK: *χ*^2^(1) = 23.403, ***p* < 0.001**
No	1284 (80.4)	495 (76.6)	375 (87.0)	414 (79.8)	
I do not know	126 (7.4)	66 (9.8)	35 (8.1)	25 (4.1)	

Number of respondents (N,n) and analyses with inferential statistics were calculated with unweighted data. Proportions were calculated with weighted data; rounding errors lead to some differences between rounded-off numerical values and actual values. *Pearson Chi-Square Test. Significant p-values are highlighted in bold.

### 3.4 Dog and cat owners’ beliefs about the use of internet resources to obtain medical information

The majority of the owners in all three countries agreed that obtaining information from the internet enables them to have a more informed discussion with their veterinarian (65.9%) and helps them to make the right decision for their animal (64.9%) (Table 4). On the question of the impact of online information on their expectations and impressions of the standard of veterinary medicine, around 40% (averaged across countries) agreed that the use of internet resources increases their expectations of the care available for their pet, but that it can also give the ‘wrong’ impression of standard veterinary medicine.

**Table 4:** Dog and Cat owners’ beliefs about the use of internet resources to find veterinary medical information.

Nr.	The use of internet resources…	Level of agreement^*^	All countries (*N* = 1606)	Austria (*n* = 657)	Denmark (*n* = 432)	United Kingdom (*n* = 517)	Test^**^
1	… increases my expectations of the standard of veterinary care available for my pet.	Disagreement	278 (16.8)	143 (20.6)	66 (15.6)	69 (13.2)	H(2) = 29.884, ***p* < 0.001**^a^ AT vs. DK: *p* = 0.600 AT vs. UK: ***p* < 0.001** DK vs. UK: ***p* < 0.001**
		Neutral	617 (38.1)	240(36.4)	196 (45.0)	181 (34.6)	
		Agreement	653 (41.4)	262 (41.1)	138 (31.9)	253 (49.3)	
		I do not know	58 (3.7)	12 (1.9)	32 (7.5)	14 (2.9)	
		Mean ± Std.	**4.39 ± 1.36**	**4.27 ± 1.39**	**4.22 ± 1.26**	**4.67 ± 1.37**	
2	… enables me to have a more informed discussion with my vet.	Disagreement	116 (6.9)	58 (8.4)	34 (8.2)	24 (4.2)	H(2) = 13.511, ***p* < 0.001**^a^ AT vs. DK: *p* = 0.293 AT vs. UK: ***p* < 0.001** DK vs. UK: ***p* < 0.001**
		Neutral	389 (24.1)	190 (28.9)	108 (24.6)	91 (17.9)	
		Agreement	1051 (65.9)	397 (61.0)	275 (63.5)	379 (73.4)	
		I do not know	50 (3.1)	12 (1.7)	15 (3.7)	23 (4.5)	
		Mean ± Std.	**5.06 ± 1.25**	**4.91 ± 1.22**	**4.92 ± 1.30**	**5.36 ± 1.2**	
3	… can lead to situations where I am better informed than my vet.	Disagreement	578 (35.1)	250 (37.1)	192 (45.0)	136 (25.0)	H(2) = 82.793, ***p* < 0.001**^a^ AT vs. DK: ***p* < 0.001** AT vs. UK: ***p* < 0.001** DK vs. UK: ***p* < 0.001**
		Neutral	449 (28.2)	186 (29.5)	137 (31.6)	126 (24.0)	
		Agreement	520 (32.8)	207 (31.3)	82 (18.4)	231 (46.0)	
		I do not know	59 (3.8)	14 (2.2)	21 (5.0)	24 (4.9)	
		Mean ± Std.	**3.97 ± 1.63**	**3.91 ± 1.55**	**3.40 ± 1.49**	**4.49 ± 1.66**	
4	… enables me to challenge my vet to justify their recommendations.	Disagreement	370 (22.3)	166 (24.6)	136 (31.4)	68 (12.4)	H(2) = 84.846, ***p* < 0.001**^a^ AT vs. DK: ***p* < 0.001** AT vs. UK: ***p* < 0.001** DK vs. UK: ***p* < 0.001**
		Neutral	514 (32.4)	220 (34.5)	144 (33.9)	150 (28.9)	
		Agreement	678 (42.3)	260 (39.3)	131 (29.5)	187 (56.0)	
		I do not know	59 (2.9)	11 (1.6)	21 (5.2)	12 (2.8)	
		Mean ± Std.	**4.31 ± 1.47**	**4.23 ± 1.47**	**3.82 ± 1.45**	**4.77 ± 1.34**	
5	… helps me to make the right decision for my animal.	Disagreement	126 (7.6)	66 (9.9)	37 (8.7)	23 (4.1)	H(2) = 53.674, ***p* < 0.001**^a^ AT vs. DK: *p* = 0.513 AT vs. UK: ***p* < 0.001** DK vs. UK: ***p* < 0.001**
		Neutral	401 (25.2)	187 (29.6)	121 (27.3)	93 (18.2)	
		Agreement	1042 (64.9)	397 (59.5)	257 (60.1)	388 (75.0)	
		I do not know	37 (2.3)	7 (0.9)	17 (3.9)	13 (2.6)	
		Mean ± Std.	**5.00 ± 1.24**	**4.79 ± 1.26**	**4.88 ± 1.21**	**5.35 ± 1.16**	
6	…enables me to buy some medication(s) more cheaply (e.g., flea treatments, wormers).	Disagreement	202 (11.7)	114 (16.0)	59 (13.4)	29 (5.4)	H(2) = 62.597, ***p* < 0.001**^a^ AT vs. DK: *p* = 0.762 AT vs. UK: ***p* < 0.001** DK vs. UK: ***p* < 0.001**
		Neutral	425 (27.0)	178 (28.2)	128 (29.6)	119 (23.7)	
		Agreement	903 (56.4)	350 (53.5)	196 (45.6)	357 (68.6)	
		I do not know	76 (4.8)	15 (2.4)	49 (11.5)	12 (2.3)	
		Mean ± Std.	**4.86 ± 1.43**	**4.66 ± 1.41**	**4.66 ± 1.51**	**5.25 ± 1.29**	
7	… can give the wrong impression of standard veterinary medicine.	Disagreement	199 (11.8)	87 (12.7)	53 (12.2)	59 (10.3)	H(2) = 21.887, ***p* < 0.001**^a^ AT vs. DK: ***p* = 0.007** AT vs. UK: ***p* = 0.016** DK vs. UK: ***p* < 0.001**
		Neutral	620 (38.8)	156 (39.4)	185 (42.6)	179 (35.2)	
		Agreement	671 (42.0)	290 (44.5)	130 (29.7)	251 (49.0)	
		I do not know	116 (7.4)	24 (3.4)	64 (15.5)	28 (5.5)	
		Mean ± Std.	**4.58 ± 1.27**	**4.56 ± 1.25**	**4.32 ± 1.23**	**4.79 ± 1.29**	

Number of respondents (N,n) and analyses with inferential statistics were calculated with unweighted data. Proportions and mean ± std were calculated with weighted data; rounding errors lead to some differences between rounded-off numerical values and actual values. *Disagreement = 1 “strongly disagree,” 2 “disagree” and 3 “somewhat disagree”; Neutral = 4 “neutral (neither agree nor disagree)”; Agreement = 5 “somewhat agree,” 6 “agree,” 7 “strongly agree”. **Kruskal-Wallis H test (answer option “I do not know” was excluded from these analyses). ^a^Bonferroni correction was applied for significant results; as there are seven tests being made one for each type of internet resource used to find medical information, alpha was divided by 7 (*n* = 7): 0.05/7 = 0.007, i.e., each test is tested against a level of 0.007. Significant p-values are highlighted in bold.

Considering differences between countries, results show that United Kingdom owners were more likely to agree with all six statements related to the use of internet resources compared to Austrian and Danish owners. The following differences can be highlighted: United Kingdom owners (46.0%) were more likely to agree that the use of internet resources can lead to situations where they are better informed than their veterinarian, and that such resources enable them to challenge their veterinarian to justify their recommendations (56.0%) compared to Austrian (31.3%; *p* < 0.001; 39.3%; *p* < 0.001) and Danish owners, respectively, (18.4%, *p* < 0.001; 29.5%, *p* < 0.001). Further, Danish owners were less likely to agree that they are better informed (*p* < 0.001) or enabled to challenge their veterinarian (*p* < 0.001) using information obtained from the internet, compared to Austrian owners.

### 3.5 What explains whether owners use internet resources to find medical information?

In all three countries, younger owners and owners who were more attached to their animal were more likely to make use of internet resources compared to older (p_AT,DK,UK_ < 0.001) and less attached animal owners (p_AT_ = 0.006; p_DK_ = 0.05;p_UK_ < 0.001) (Supplementary Table 2). Further, Danish dog owners were more likely to make use of internet resources compared to Danish cat owners (*p* = 0.039). United Kingdom owners who work in the veterinary field were more likely to make use of the internet compared to owners who did not (*p* = 0.012).

### 3.6 What explains how often owners use internet resources prior to a consultation with their veterinarian?

In all three countries, owners who were more likely to agree that the use of internet resources enables them to have a more informed discussion with their veterinarians more often make use of them prior to a consultation (p_AT_ < 0.001; p_DK_ = 0.002; p_UK_ = 0.030) (Supplementary Table 3). Again, in all three countries, younger owners more often make use of internet resources prior to a consultation compared to older owners (p_AT_ < 0.001; p_DK_ = 0.002; p_UK_ = 0.001). Further, Danish owners who were more likely to agree that the use of internet resources helps them to challenge their veterinarian to justify their recommendation more often make use of internet resources prior to a consultation (*p* = 0.049). In the UK, male owners were more likely to use internet resources prior to a consultation compared to female owners (*p* < 0.001).

### 3.7 What explains how often owners use internet resources after a consultation with their veterinarian?

In all three countries, owners who were more likely to agree that the use of internet resources helps them to make the right decision more frequently make use of internet resources after a consultation (p_AT_ < 0.001; p_DK_ = 0.013; p_UK_ < 0.001) (Supplementary Table 4). Further, younger Austrian, Danish and United Kingdom owners more frequently make use of internet resources after a consultation compared to older owners (p_AT,UK_ < 0.001; p_DK_ = 0.002). For Denmark, owners who work in the veterinary field make more use of internet resources after a consultation compared to owners who do not (*p* = 0.013). Further, for the United Kingdom, male owners, cat owners and owners who are more attached to their animals more frequently make use of the internet after a consultation compared to female owners (*p* = 0.011), dog owners (*p* < 0.001) and owners who are less attached to their animal (*p* = 0.042).

## 4 Discussion

The results of this study show that although more than half of pet owners in the three countries use internet resources to find medical information, quite a few – around a third of respondents – stated that they never use them either prior to or after a consultation with their veterinarian. However, for all three countries, we identified that younger owners and owners who are more attached to their pet made more use of the internet to find information regarding companion animal health and veterinary treatments. Considering the type of internet resources, we found that websites providing veterinary information (55%), practice websites (35%) and websites of veterinary associations (24%) are used more often than social media (18.5%) or blogs and forums (11.7%). When owners were asked whether they have ever disagreed with a veterinarian’s professional advice based on information they had obtained from the internet, only a minority of respondents (12%) indicated such disagreement. Rather, across all three countries, we found that owners believe that the use of internet resources can lead to better informed discussion with their veterinarian, or help them to make the right decision for their animal. Not surprisingly, owners who felt that way more often made use of the internet to find medical information before or after a consultation with their veterinarian.

Other studies looking at the use internet resources by owners show some interesting differences. For example, in contrast to our findings that around one in three owners never use internet resources, other studies have reported that only around 6% of surveyed US owners (7) and even fewer (3%) of United Kingdom dog owners stated that they do not use the internet for pet health information (2). A possible explanation could be differences in sampling methods. Thus, Kogan and colleagues recruited respondents from two United States metropolitan areas (7), where internet usage is typically higher than in rural areas, or through social media using convenience sampling and snowballing (2). Both recruitment techniques risk a positive bias toward internet users to some extent, whereas our study is based on a representative sample of dog and cat owners, and may provide a more representative picture of owners’ use of internet resources.

Due to less frequent general daily use of the internet (10), we expected that Austrian owners would less often use the internet to search for pet health information compared with Danish and United Kingdom owners. However, our results show the opposite: we found that Austrian and United Kingdom owners significantly more often use the internet after a consultation in comparison to Danish owners. This could, as we will discuss below, potentially be explained by different levels of trust in the three countries.

On the question of when owners consult the internet, our data show that in general owners tend to make more use of the internet prior to than after a consultation with their veterinarian. This may be because owners are seeking information to help them decide whether they need to make an appointment with the veterinarian or not, which was also identified as a reason for online searches in the study by Kogan et al. (7).

Further, our study reveals that younger owners in particular make use of internet resources to find medical information, in agreement with Kogan et al. (7), who found that younger US owners (20–30 years) were more likely to use the internet for pet health information than other age groups. This does not seem surprising as, for example, a study on internet use in Europe showed that in 2019, 94% of people aged 25 to 34 used the internet daily, compared to only 69% of people aged 55 to 64 (18). Considering owners’ close emotional attachment to their pet(s), we assumed that a strong attachment would increase the likelihood that owners would search the internet for medical information. Our results support this, in agreement with other studies showing that owners’ close emotional bond increases their interest in and expectations from veterinary health care (4, 19, 20). Utilizing internet resources can help fulfill owners’ needs by providing valuable information on various veterinary care options.

With respect to possible differences between dog and cat owners in relation to the use of internet resources in the veterinary context, we found some clear country differences. Whereas Danish dog owners are more likely to make use of internet resources compared to cat owners, United Kingdom cat owners more frequently use internet resources after a consultation with their veterinarian. These differences might be explained by findings of a comparative study on how much owners care for their cats or dogs (21). In the latter study, differences in caring for cats vs. dogs were rather modest in the United Kingdom, in contrast to Denmark, where a large difference was identified, with Danish owners caring far more about their dogs than cats. This may explain why more Danish dog owners make use of the internet to search for information compared to cat owners. However, it could equally be argued that if owners do not care as much about the animal, e.g., their cat, they would be less likely to be willing to spend money seeing a veterinarian, and might first try to find the information on the internet. With the rising costs for veterinary healthcare services, future research is merited into the relationship between the use of internet resources and owners’ financial concerns.

When owners do make use of the internet, different types of information sources may be chosen. Here we found clear differences between the three countries. Significantly more United Kingdom dog and cat owners (26%) make use of social media to find medical information on pets, while only 16% of Austrian and 12% of Danish owners use this information source. The greater use of social media in the UK to search for pet health information reflects the findings of Kuhl et al. (1) who collected data among dog owners in 2013 and found that over 20% of United Kingdom dog owners used social networking sites. We also found that significantly more Danish owners than Austrian and British owners use websites providing veterinary information or practice websites of (their) veterinarians. As we have not specified the source of the websites providing veterinary information (these may vary widely in terms of the information provided and the trustworthiness of the source), the results should be treated with caution. However, turning to the use of practice websites, we believe that the use of this as a source of information for owners is an interesting result and in particular of value for veterinarians. In contrast to general websites, the content of a veterinary practice website can be curated by the veterinary professionals to ensure only accurate and trustworthy information is put online. As such, veterinarians should ensure their practice websites are optimized and regularly updated to provide relevant information and/or provide links to direct owners to other trustworthy websites.

Another aim of the study was to identify whether owners have ever disagreed with a veterinarian’s professional advice based on information they had obtained from the internet. Based on what is already known about differences in national levels of general trust, we expected that Danish owners in particular would have a higher level of trust in the professional advice of veterinarians, and be less likely to disagree with that advice. Our results supported this assumption, as significantly fewer (4.9%) Danish owners reported that they ever disagreed with their veterinarian’s advice, compared to Austrian (13.6%) or United Kingdom (16.1%) owners. Similarly, significantly more Danish owners disagreed that the use of internet resources enables them to challenge their veterinarian to justify the professional recommendations. This may be closely linked to the finding – as highlighted above – that almost half of Danish owners use veterinary practice websites where one can assume that the information resource is of high quality and matches the veterinarians’ advice. Danish owners who were more likely to agree that the use of internet resources helps them to challenge their veterinarian to justify their recommendation more often made use of the internet prior to consultation with their veterinarian. This may be because such owners have previously found that their veterinarians have not always provided all the available information to them. As such, having more information before a consultation can lead to better informed discussions with the veterinarian from the owners’ perspective. This can be further supported by the result that owners who were more likely to agree that the use of internet resources enables them to have a more informed discussion with their veterinarians more often make use of these resources prior to a consultation.

Whereas consulting the internet before a consultation seems to enable owners to have more informed discussions with their veterinarians, owners who used the internet after the consultation were more likely to agree that it helps them to make right decisions. Modern advancements in small animal practice have meant that owners are often faced with making challenging decisions on a wide range of diagnostic and treatment options, and information gained from internet resources can provide additional support and increased clarity in this regard. This is supported by findings of Kogan et al. (22), who found that around 55% of Australian pet owners surveyed (strongly) agreed that online pet health information influenced their decision about how to treat their pet’s illness or condition, and 73% (strongly) agreed that online information helped them to make better choices about their pet’s health. Interestingly, we identified that whether or not owners work in the veterinary field (e.g., practicing vets, veterinary nurses, veterinary assistants) influenced whether and how often they use the internet to find information. In the United Kingdom, such owners were more inclined to look up information online. Similarly, in Denmark, veterinary professionals tended to use the internet more often after a consultation compared to non-veterinary professionals. This is perhaps unsurprising, as those working in the veterinary sector are likely to be familiar with using the internet to search for relevant information, and also more aware of the need for specialist knowledge.

Since the present study was developed in light of a previous survey providing empirical insights into veterinarians’ attitudes toward clients’ use of online resources (3), to the best of the authors’ knowledge, this is the first time that veterinarians and owners’ perspectives in the three countries studied can be directly compared on this issue. Interestingly, while over 60% of owners reported that they use internet resources prior to a consultation with their veterinarian, only around 20% of the surveyed veterinarians estimated that around that number of owners (60–79%) would do so (3). Although the owner data was collected 2 years later than the data from the veterinarians, it seems unlikely that internet use and the availability of websites has increased dramatically during this time, and it seems more likely that the use of online information is not openly discussed between the veterinarians and their clients.

Open discussion can have a positive effect on patient care, and the use of online resources by clients can impact the dynamics of such discussions about diagnostics and treatments during consultations. In relation to this, we identified interesting differences between veterinarians and owners. Whereas over 70% of surveyed veterinarians agreed that clients’ use of internet resources results in greater expectations of advanced diagnostics and treatments and causes clients to question their recommendations (3), only around 40% of dog and cat owners agreed with these potential effects. A difference was also observed in regard to the question of whether the use of online resources improves discussions among veterinarians and their clients. Fewer veterinarians (49%) agreed with this potential benefit (3), compared to 66% owners who felt that it would improve discussion from their perspective. Against the background of these differences, it can be argued that clients may gain a level of self-assurance by using online information, which they feel benefits them during consultations. Although professionals may sometimes feel challenged and uncomfortable during these discussions based on online information, we believe that veterinarians should take the opportunity to promote an open conversation on the use of online information, and, importantly, direct owners to trustworthy sources. Such open conversations paired with the provision of appropriate information on the practice websites, including links to other useful websites, can help to eliminate misinformation and possible mistrust about the best patient care, and help to strengthen the relationship between the veterinarian and the client.

## 5 Conclusions and practical implications for veterinarians

The findings of this study have some important practical implications for veterinarians. In our view there are three ways in which veterinarians can respond to the increasing use of online resources by their clients and the discussions that arise as a result during consultations.

Perhaps our most important finding is that, although veterinarians often think that clients want to challenge them based on information they have obtained online, we found that only a small percentage of pet owners actually do this. Instead, the aim of most clients is to be better prepared for the consultation with the veterinarian or to find support in making the right decision afterwards. As having better informed owners can lead to improved discussions and decision-making with their veterinarians, we encourage veterinarians to view this trend in a positive and constructive way.

Secondly, in cases where a client has obtained information from the internet that contradicts the veterinarian’s advice, it may be optimal for the veterinarian to start with an open and transparent discussion about the online searches made by the client. Such an open discussion can have a positive effect on both the client’s subsequent decision-making, and in building trust between the client and the veterinarian.

Thirdly, we found that more than a third of pet owners consult veterinary practice websites to obtain information. In light of this, we encourage veterinarians to invest in developing websites that not only advertise the veterinary services that the practice provides, but that also provide high quality evidence-based veterinary medical information, or provide links to other such websites, that their clients can consult.

### 5.1 Study limitations

Although this representative study includes three countries to provide a comprehensive investigation of dog and cat owners’ use of and beliefs about internet resources in the veterinary context, the study is subject to several limitations. First, as already indicated, interpretation with respect to the use of veterinary medical websites should be treated with caution as we did not define exactly what constitutes a ‘veterinary medical website’. Second, since practice websites as well as websites of veterinary associations are both online resources providing medical information, these are not clearly distinct categories. Third, the use of answer options such as “occasionally” or “frequently” is more subjective compared to, for example, stating a specific percentage, and so respondents may have interpreted these terms differently, introducing variability into the reported rates of internet use.

## Data availability statement

The original contributions presented in the study are included in the article/Supplementary material, further inquiries can be directed to the corresponding authors.

## Ethics statement

The studies involving humans were approved by Research Ethics Committee of SCIENCE and HEALTH at the University of Copenhagen. The studies were conducted in accordance with the local legislation and institutional requirements. The participants provided their written informed consent to participate in this study.

## Author contributions

SS: Conceptualization, Data curation, Formal analysis, Investigation, Methodology, Project administration, Software, Visualization, Writing – original draft. TL: Conceptualization, Data curation, Methodology, Project administration, Validation, Writing – review & editing. SC: Conceptualization, Project administration, Writing – review & editing. PS: Conceptualization, Funding acquisition, Project administration, Writing – review & editing.
