# Supplementary material for: Does “Dr. Google” improve discussion and decisions in small animal practice? Dog and cat owners use of internet resources to find medical information about their pets in three European countries
Source: Front Vet Sci. 2024 Jun 19;11:1417927. doi: 10.3389/fvets.2024.1417927 (PMC11223573; doi:10.3389/fvets.2024.1417927)
Supplement: Supplementary file 1 [file Table_1.docx]

Supplementary Table 1

Socio-demographic factors and animal related aspects

|  | **All**  **(N=2117)^+^** | **Austria**  **(n=800)^+^** | **Denmark**  **(n=626)^+^** | **UK**  **(n=691)^+^** | **Test** |
| --- | --- | --- | --- | --- | --- |
| **Age (years)** | | | |  |  |
| Median [IQR] | 47 [32;59] | 47 [32;59] | 48 [35;59] | 45 [30;58] |  |
| **Age groups (years)** | | | |  | *Kruskal-Wallis H tests* |
| 18-30 | 495 (21.9) | 174 (22.4) | 107 (17.4) | 178 (25.3) | AT vs DK: **p=0.014**  AT vs UK: p=0.617  DK vs UK: **p<0.001** |
| 31-40 | 356 (17.0) | 130 (16.6) | 97 (15.8) | 130 (18.4) |  |
| 41-50 | 385 (18.4) | 129 (16.6) | 137 (22.3) | 119 (16.9) |  |
| 51-60 | 434 (20.7) | 176 (22.5) | 128 (20.9) | 131 (18.5) |  |
| <60 | 461 (22.0) | 171 (21.9) | 145 (23.6) | 146 (20.8) |  |
| **Gender** | | | |  | *Chi-square test* |
| Male | 964 (46.0) | 357 (45.7) | 272 (44.3) | 336 (47.7) | p=0.431 |
| Female | 1125 (53.6) | 419 (53.8) | 341 (55.5) | 365 (51.8) |  |
| Neither of these | 8 (0.4) | 4 (0.5) | 1 (0.2) | 3 (0.4) |  |
| **Gross household income** | | | | | *Kruskal-Wallis H tests* |
| I don’t know / prefer not to say | | 132 (17.0) | 92 (14.9) | 68 (9.7) |  |
| Low^*^ | | 207 (26.6) | 131 (21.3) | 185 (26.3) | AT vs. DK: **p=0.015**  AT vs UK: p=0.626  Dk vs UK: **p<0.001** |
| Middle^**^ | | 243 (31.1) | 174 (28.3) | 225 (32.0) |  |
| High^***^ | | 197 (25.3) | 217 (35.4) | 225 (32.0) |  |
| **Health insurance** | | | | | *Chi-square test* |
| yes | 874 (41.7) | 166 (21.3) | 346 (56.3) | 362 (51.5) | AT vs DK: **p<0.001**  AT vs UK: **p<0.001**  DK vs UK: p=0.078 |
| No/not anymore | 1223 (58.3) | 614 (78.7) | 268 (43.7) | 341 (48.5) |  |
| **Species** | | | | | *Chi-square test* |
| Dog | 847 (40.4) | 223 (28.6) | 305 (49.7) | 319 (45.3) | **p<0.001** |
| Cat | 860 (41.0) | 383 (49.1) | 236 (38.5) | 241 (34.2) |  |
| Both | 391 (18.5) | 174 (22.3) | 73 (11.9) | 144 (20.5) |  |
| **Species** *(only dog and cat owner based on answers provide the LAPS)* | | | | | *Chi-square test* |
| Dog | 1132 (55.3) | 355 (46.7) | 367 (60.3) | 409 (60.5) | **p<0.001** |
| Cat | 915 (43.6) | 406 (53.3) | 242 (39.7) | 267 (39.5) |  |
| **Visits to veterinarian in the last twelve months (for dog owners)** | | | | |  |
| Mean ± Std. | 2.9 ± 4.02 | 3.41 ± 4.37 | 2.21 ± 2.02 | 3.08 ± 4.78 |  |
| Median [IQR] | 2.0 [1;3] | 2 [1;4] | 2 [1;3] | 2 [1;3] |  |
| **Visits to veterinarian in the last twelve months (for cat owners)** | | | | |  |
| Mean ± Std. | 2.4±4.48 | 2.86 ± 5.35 | 1.54 ± 1.56 | 2.46 ± 4.59 |  |
| Median [IQR] | 1 [0.5;2.0] | 1 [0.5;3] | 1 [0.5;2] | 1 [0.5;2] |  |
| **Do you live alone?** | | | | | *Chi-square test* |
| yes | 447 (21.3) | 180 (23.1) | 130 (21.1) | 136 (19.4) | p=0.251 |
| no | 1650 (78.8) | 600 (76.9) | 483 (78.8) | 567 (80.6) |  |

*^+^ Number of respondents (n) calculated with unweighted data*

*^*^AT: less than 13450 – 26899 €; DK: less than 100.000 – 300.000 DK; UK: less than 11200 – 22399 GBP*

*^**^AT: 26900 – 53799 €; DK: 300.001 – 600.00 DK; UK: 22400 – 44799 GBP*

*^***^AT: 53800 – more than 134500 €; DK: 600.001 – more than 1.000.001 DK; UK: 44800 – more than 112000 GBP*
