# Supplementary material for: Does “Dr. Google” improve discussion and decisions in small animal practice? Dog and cat owners use of internet resources to find medical information about their pets in three European countries
Source: Front Vet Sci. 2024 Jun 19;11:1417927. doi: 10.3389/fvets.2024.1417927 (PMC11223573; doi:10.3389/fvets.2024.1417927)
Supplement: Supplementary file 2 [file Table_2.docx]

Supplementary Table 2

Binary logistic regression analyses of socio-demographic aspects, animal-related aspects and owner’s emotional attachment to the animal towards owners’ use of internet resources to obtain medical information

| **Model Austria**: (χ^2^(6)=40.818, **p<0.001**) | | | | | |
| --- | --- | --- | --- | --- | --- |
|  | **B** | **Std. Error** | **Wald Chi-Square** | **df** | **Sig.** |
| **Emotional attachment** (LAPS mean) | 0.542 | 0.198 | 7.518 | 1 | **0.006** |
| **Animal species**  *(Ref. cat.: Cat)* | -0.161 | 0.219 | 0.539 | 1 | 0.463 |
| **Gender**  *(Ref. cat.: Female)* | 0.203 | 0.223 | 0.827 | 1 | 0.363 |
| **Age** | -0.032 | 0.006 | 25.774 | 1 | **<0.001** |
| **Work in veterinary field**  *(Ref. cat.: No)* | -0.120 | 0.471 | 0.065 | 1 | 0.799 |
| **Living alone**  *(Ref. cat.: No)* | -0.369 | 0.257 | 2.073 | 1 | 0.150 |
| **Model Denmark**: (χ^2^(6)=55.951, **p<0.001**) | | | | | |
|  | **B** | **Std. Error** | **Wald Chi-Square** | **df** | **Sig.** |
| **Emotional attachment** (LAPS mean) | 0.357 | 0.182 | 3.840 | 1 | **0.050** |
| **Animal species**  *(Ref. cat.: Cat)* | -0.405 | 0.196 | 4.262 | 1 | **0.039** |
| **Gender**  *(Ref. cat.: Female)* | 0.170 | 0.200 | 0.728 | 1 | 0.394 |
| **Age** | -0.037 | 0.006 | 37.702 | 1 | **<0.001** |
| **Work in veterinary field**  *(Ref. cat.: No)* | -1.140 | 1.078 | 1.117 | 1 | 0.291 |
| **Living alone**  *(Ref. cat.: No)* | -0.927 | 1.231 | 5.650 | 1 | **0.017** |
| **Model UK**: (χ^2^(6)=102.658, p<0.001) | | | | | |
|  | **B** | **Std. Error** | **Wald Chi-Square** | **df** | **Sig.** |
| **Emotional attachment** (LAPS mean) | 0.769 | 0.205 | 14.019 | 1 | **<0.001** |
| **Animal species**  *(Ref. cat.: Cat)* | -0.088 | 0.219 | 0.163 | 1 | 0.686 |
| **Gender**  *(Ref. cat.: Female)* | 0.021 | 0.219 | 0.009 | 1 | 0.923 |
| **Age** | -0.044 | 0.006 | 50.245 | 1 | **<0.001** |
| **Work in veterinary field**  *(Ref. cat.: No)* | -1.881 | 0.749 | 6.305 | 1 | **0.012** |
| **Living alone**  *(Ref. cat.: No)* | 0.045 | 0.259 | 0.030 | 1 | 0.826 |
