# Supplementary material for: Does “Dr. Google” improve discussion and decisions in small animal practice? Dog and cat owners use of internet resources to find medical information about their pets in three European countries
Source: Front Vet Sci. 2024 Jun 19;11:1417927. doi: 10.3389/fvets.2024.1417927 (PMC11223573; doi:10.3389/fvets.2024.1417927)
Supplement: Supplementary file 3 [file Table_3.docx]

Supplementary Table 3

Ordinal regression analyses of socio-demographic aspects, animal-related aspects, owners’ emotional attachment to the animal, and beliefs regarding the use of internet resources on the frequency of owners’ use of internet resources *prior to* consultation with their veterinarian to obtain medical information

| **Model Austria**: (χ^2^(8)=50.545, **p<0.001**) | | | | | |
| --- | --- | --- | --- | --- | --- |
|  | Hypothesis Test | | | | |
|  | **B** | **Std. Error** | **Wald Chi-Square** | **df** | **Sig.** |
| **Age** | -0.034 | 0.007 | 24.468 | 1 | **<0.001** |
| **Emotional attachment**  (LAPS mean) | 0.203 | 0.222 | 0.835 | 1 | 0.361 |
| **Owners’ belief 1:** “The use of internet resources enables me to have a more informed discussion with my vet.” | 0.329 | 0.092 | 12.798 | 1 | **<0.001** |
| **Owners’ belief 2:** “The use of internet resources enables me to challenge my vet to justify the recommendation.” | 0.069 | 0.069 | 0.986 | 1 | 0.321 |
| **Gender**  *(Ref. cat.: Female)* | 0.080 | 0.225 | 0.126 | 1 | 0.722 |
| **Work in veterinary field**  *(Ref. cat.: No)* | -0.154 | 0.387 | 0.159 | 1 | 0.690 |
| **Living alone**  *(Ref. cat.: No)* | 0.366 | 0.240 | 2.322 | 1 | 0.128 |
| **Animal species**  *(Ref. cat.: Cat)* | -0.193 | 0.212 | 0.832 | 1 | 0.362 |
| **Model Denmark**: (χ^2^(8)=38.291, **p<0.001**) | | | | | |
|  | Hypothesis Test | | | | |
|  | **B** | **Std. Error** | **Wald Chi-Square** | **df** | **Sig.** |
| **Age** | -0.024 | 0.008 | 9.336 | 1 | **0.002** |
| **Emotional attachment**  (LAPS mean) | -0.082 | 0.249 | 0.107 | 1 | 0.743 |
| **Owners’ belief 1:** “The use of internet resources enables me to have a more informed discussion with my vet.” | 0.361 | 0.114 | 9.972 | 1 | **0.002** |
| **Owners’ belief 2:** “The use of internet resources enables me to challenge my vet to justify the recommendation.” | 0.175 | 0.089 | 3.884 | 1 | **0.049** |
| **Gender**  *(Ref. cat.: Female)* | 0.005 | 0.258 | 0.000 | 1 | 0.984 |
| **Work in veterinary field**  *(Ref. cat.: No)* | 0.768 | 0.738 | 1.084 | 1 | 0.298 |
| **Living alone**  *(Ref. cat.: No)* | 0.079 | 0.291 | 0.074 | 1 | 0.786 |
| **Animal species**  *(Ref. cat.: Cat)* | 0.258 | 0.245 | 1.112 | 1 | 0.292 |
| **Model UK**: (χ^2^(8)=63.790, **p<0.001**) | | | | | |
|  | Hypothesis Test | | | | |
|  | **B** | **Std. Error** | **Wald Chi-Square** | **df** | **Sig.** |
| **Age** | -0.024 | 0.007 | 10.506 | 1 | **0.001** |
| **Emotional attachment**  (LAPS mean) | 0.417 | 0.246 | 2.866 | 1 | 0.090 |
| **Owners’ belief 1:** “The use of internet resources enables me to have a more informed discussion with my vet.” | 0.238 | 0.110 | 4.729 | 1 | **0.030** |
| **Owners’ belief 2:** “The use of internet resources enables me to challenge my vet to justify the recommendation.” | 0.106 | 0.093 | 1.285 | 1 | 0.257 |
| **Gender**  *(Ref. cat.: Female)* | 0.774 | 0.229 | 11.368 | 1 | **<0.001** |
| **Work in veterinary field**  *(Ref. cat.: No)* | 0.616 | 0.343 | 3.215 | 1 | 0.073 |
| **Living alone**  *(Ref. cat.: No)* | 0.166 | 0.295 | 0.317 | 1 | 0.574 |
| **Animal species**  *(Ref. cat.: Cat)* | -0.479 | 0.222 | 4.654 | 1 | **0.031** |
