# Supplementary material for: Does “Dr. Google” improve discussion and decisions in small animal practice? Dog and cat owners use of internet resources to find medical information about their pets in three European countries
Source: Front Vet Sci. 2024 Jun 19;11:1417927. doi: 10.3389/fvets.2024.1417927 (PMC11223573; doi:10.3389/fvets.2024.1417927)
Supplement: Supplementary file 5 [file Data_Sheet_1.PDF]

Introduction and background:

Thank you for participating in this survey.

The **purpose** of this survey is to **explore why some people keep pets and others do not, and to explore the attitudes of pet owners to developments in modern small animal practice.**
The **survey will take approximately 3 minutes** to complete for **non-pet owners**. **If you have a pet**, we will also ask about **your attachment to your pet** (or pets), and your **attitude towards modern veterinary practice** and **expectations of veterinary services**. There are also some questions about health insurance for pets, as well as the use of social media and internet resources in relation to veterinary treatment. For **pet owners, the questionnaire will take approximately 15- 20 minutes** to complete.

This questionnaire is from a joint research project with three involved countries: Denmark, Austria, and the UK. The respective researchers are from the University of Copenhagen, University of Veterinary Medicine, Vienna, and the University of Glasgow.

Completion of the questionnaire is **voluntary,** and you can **exit at any point prior to submitting the final answer**. Your responses will then be passed to the researchers in an **anonymized** form, and **no information can be traced back to you**.

When clicking on the button "Next", you confirm that you are over 17 years old, and consent to participate in this survey.

Thank you for your contribution!

SECTION A:
Information on your pet, demographics, and the veterinary practice/clinic you attend.

Instruction A.1: single-choice question

A.1: Pets are often kept at home, but we also want to hear about animals that are not kept at home (e.g. horses). However, we are not interested here in production animals kept on farms e.g. dairy cows.
Do you have one or more pet(s)?

Yes	
No	

Instruction A.2: multiple choice question and only if A1=Yes

A.2: How many of the following pets do you have? Please indicate the number of each type, or select 0 (none) where appropriate.

	0 (none)	1	2	3	4	More than 4
Dog						
Cat						
Horse						
Rabbit						
Rodent (e.g. hamster, guinea pig, chinchilla, mouse/rat)						
Bird						
Reptile (e.g. lizard, snake, turtle)						
Fish (both aquarium fish and fish in garden pond)						
Other pet than the above mentioned						

Construct filter variable = ***Number of pet species***

Count = ***Number of pet species*** *if A2Dog>0, A2Cat>0, A2Horse>0, A2Rabbit>0, A2Rodent>0, A2Bird>0, A2Reptile>0, A2Fish>0, A2*Other pet than the above mentioned *>0*

Instruction A.3.1: only if A2Dog=1

A.3.1: Please provide the age of your dog, the number of visits to a veterinarian in the last 12 months, and whether the dog is covered by a health insurance.

Scroll down menu: age (<0.5 - >20 years)
Scroll down menu: number of visits to a veterinarian in the last 12 months (0 - > 20)
Scroll down menu: health insurance (yes, no, not anymore)

Instruction A.3.1.1: English questionnaire: single-choice question and only if A3.1Health insurance=Yes

A.3.1.1: What type of insurance do you have for your dog?

scroll-down menu (A policy where the limit is *per year,* A policy where the limit is *per condition, I don't know)*

Instruction A.3.1.2: single-choice question and only if A.3.1.1=A policy where the limit is *per year*.

A.3.1.2: What is the insurance limit for your dog?

scroll-down menu (I don't know, up to £1500 per year, between £1501- £4000 per year, between £4001- £8000 per year, more than £8000 per year)

Instruction A.3.1.3: single-choice question and only if A.3.1.1=A policy where the limit is *per condition*.

A.3.1.3: What is the insurance limit for your dog?

scroll-down menu (I don't know, up to £1500 per condition, between £1501- £4000 per condition, between £4001- £8000 per condition, more than £8000 per condition)

Instruction A.3.2: only if A2Dog=2

A.3.2: Please provide the age of your dogs, the number of visits to a veterinarian in the last 12 months, and whether the two dogs are covered by health insurance.

A.3.2.1:

Scroll down menu: age (<0.5 - >20 years)
Scroll down menu: number of visits to a veterinarian in the last 12 months (0 - > 20)
Scroll down menu: health insurance (yes, no, not anymore)

A.3.2.2:

Scroll down menu: age (<0.5 - >20 years)
Scroll down menu: number of visits to a veterinarian in the last 12 months (0 - > 20)
Scroll down menu: health insurance (yes, no, not anymore)

Instruction A.3.3: only if A2Dog=3

A.3.3: Please provide the age of your dogs, the number of visits to a veterinarian in the last 12 months, and whether the three dogs are covered by health insurance.

A.3.3.1:

Scroll down menu: age (<0.5 - >20 years)
Scroll down menu: number of visits to a veterinarian in the last 12 months (0 - > 20)
Scroll down menu: health insurance (yes, no, not anymore)

A.3.3.2:

Scroll down menu: age (<0.5 - >20 years)
Scroll down menu: number of visits to a veterinarian in the last 12 months (0 - > 20)
Scroll down menu: health insurance (yes, no, not anymore)

A.3.3.3:

Scroll down menu: age (<0.5 - >20 years)
Scroll down menu: number of visits to a veterinarian in the last 12 months (0 - > 20)
Scroll down menu: health insurance (yes, no, not anymore)

Instruction A.3.4: only if A2Dog>3
A.3.4: We will now ask about the age of three of your dogs, the number of visits to a veterinarian in the last 12 months, and whether the three dogs are covered by health insurance. Please choose the three dogs whose names come first in the alphabet.

A.3.4.1: First dog (first letter in name comes first in the alphabet)
Scroll down menu: age (<0.5 - >20 years)
Scroll down menu: number of visits to a veterinarian in the last 12 months (0 - > 20)
Scroll down menu: health insurance (yes, no, not anymore)

A.3.4.2: Second dog (first letter in name comes second in the alphabet)
Scroll down menu: age (<0.5 - >20 years)
Scroll down menu: number of visits to a veterinarian in the last 12 months (0 - > 20)
Scroll down menu: health insurance (yes, no, not anymore)

A.3.4.3: Third dog (first letter in name comes third in the alphabet)
Scroll down menu: age (<0.5 - >20 years)
Scroll down menu: number of visits to a veterinarian in the last 12 months (0 - > 20)
Scroll down menu: health insurance (yes, no, not anymore)

Instruction A.3.5: English questionnaire: only if A.3.2.1Health insurance=Yes or A.3.2.2Health insurance=Yes or A.3.3.1Health insurance=Yes or A3.3.2Health insurance=Yes or A3.3.3Health insurance=Yes or A.3.4.1Health insurance=Yes or A3.4.2Health insurance=Yes or A3.4.3Health insurance=Yes

A.3.5: What type of insurance do you have for your dog(s)? *If the type of insurance is not the same for all dogs, then think about the dog whose name comes first in the alphabet.*
Scroll-down menu (A policy where the limit is *per year,* A policy where the limit is *per condition, I don't know)*

Instruction A.3.5.1: single-choice question and only if A.3.5 =A policy where the limit is *per year*.
A.3.5.1: What is the insurance limit for your dog(s)? *If the limit is not the same for all dogs, then think about the dog whose name comes first in the alphabet.*

Scroll-down menu (I don't know, up to £1500 per year, between £1501- £4000 per year, between £4001- £8000 per year, more than £8000 per year)

Instruction A.3.5.2: single-choice question and only if A.3.5 =A policy where the limit is *per condition*.
A.3.5.2: What is the insurance limit for your dog(s)? *If the limit is not the same for all dogs, then think about the dog whose name comes first in the alphabet.*
Scroll-down menu (I don't know, up to £1500 per condition, between £1501- £4000 per condition, between £4001- £8000 per condition, more than £8000 per condition)

Instruction A.3.6: only if A2Cat=1
A.3.6: Please provide the age of your cat, the number of visits to a veterinarian in the last 12 months, and whether the cat is covered by health insurance.

Scroll down menu: age (<0.5 - >20 years)
Scroll down menu: number of visits to a veterinarian in the last 12 months (0 - > 20)
Scroll down menu: health insurance (yes, no, not anymore)

Instruction A.3.6.1: English questionnaire: single-choice question and only if A3.6Health insurance=Yes

A.3.6.1: What type of insurance do you have for your cat?
scroll-down menu (A policy where the limit is *per year,* A policy where the limit is *per condition, I don't know)*

Instruction A.3.6.2: single-choice question and only if A.3.6.1 =A policy where the limit is *per year*.
A.3.6.2: What is the insurance limit for your cat?
Scroll-down menu (I don't know, up to £1500 per year, between £1501- £4000 per year, between £4001- £8000 per year, more than £8000 per year)

Instruction A.3.6.3: single-choice question and only if A.3.6.1 =A policy where the limit is *per condition*.
A.3.6.3: What is the insurance limit for your cat?
Scroll-down menu (I don't know, up to £1500 per condition, between £1501- £4000 per condition, between £4001- £8000 per condition, more than £8000 per condition)

Instruction A.3.7: only if A2Cat=2
A.3.7: Please provide the age of your cats, the number of visits to a veterinarian in the last 12 months, and whether the two cats are covered by health insurance.

A.3.7.1:
Scroll down menu: age (<0.5 - >20 years)
Scroll down menu: number of visits to a veterinarian in the last 12 months (0 - > 20)
Scroll down menu: health insurance (yes, no, not anymore)

A.3.7.2:
Scroll down menu: age (<0.5 - >20 years)
Scroll down menu: number of visits to a veterinarian in the last 12 months (0 - > 20)
Scroll down menu: health insurance (yes, no, not anymore)

Instruction A3.8: only if A2Cat=3
A.3.8: Please provide the age of your cats, the number of visits to a veterinarian in the last 12 months, and whether the three cats are covered by health insurance.

A.3.8.1:
Scroll down menu: age (<0.5 - >20 years)
Scroll down menu: number of visits to a veterinarian in the last 12 months (0 - > 20)
Scroll down menu: health insurance (yes, no, not anymore)

A.3.8.2:
Scroll down menu: age (<0.5 - >20 years)
Scroll down menu: number of visits to a veterinarian in the last 12 months (0 - > 20)
Scroll down menu: health insurance (yes, no, not anymore)

A.3.8.3:
Scroll down menu: age (<0.5 - >20 years)
Scroll down menu: number of visits to a veterinarian in the last 12 months (0 - > 20)
Scroll down menu: health insurance (yes, no, not anymore)

Instruction A.3.9: only if A2Cat>3
A.3.9: We will now ask about the age of three of your cats, the number of visits to a veterinarian in the last 12 months, and whether the three cats are covered by health insurance. Please choose the three cats whose names come first in the alphabet.

A.3.9.1: First cat (first letter in name comes first in the alphabet)
Scroll down menu: age (<0.5 - >20 years)
Scroll down menu: number of visits to a veterinarian in the last 12 months (0 - > 20)

Scroll down menu: health insurance (yes, no, not anymore)

A.3.9.2: Second cat (first letter in name comes second in the alphabet)
Scroll down menu: age (<0.5 - >20 years)
Scroll down menu: number of visits to a veterinarian in the last 12 months (0 - > 20)
Scroll down menu: health insurance (yes, no, not anymore)

A.3.9.3: Third cat (first letter in name comes third in the alphabet)
Scroll down menu: age (<0.5 - >20 years)
Scroll down menu: number of visits to a veterinarian in the last 12 months (0 - > 20)
Scroll down menu: health insurance (yes, no, not anymore)

Instruction A.3.10: English questionnaire: only if A.3.7.1Health insurance=Yes or A.3.7.2Health insurance=Yes or A.3.8.1Health insurance=Yes or A3.8.2Health insurance=Yes or A3.8.3Health insurance=Yes or A.3.9.1Health insurance=Yes or A3.9.2Health insurance=Yes or A3.9.3Health insurance=Yes
A.3.10: What type of insurance do you have for your cat(s)? *If the type of insurance is not the same for all cats, then think about the cat whose name comes first in the alphabet.*
Scroll-down menu (A policy where the limit is *per year,* A policy where the limit is *per condition, I don't know)*

Instruction A.3.10.1: single-choice question and only if A.3.10=A policy where the limit is *per year*.
A.3.9.1: What is the insurance limit for your cat(s)? *If the limit is not the same for all cats, then think about the cat whose name comes first in the alphabet.*
Scroll-down menu (I don't know, up to £1500 per year, between £1501- £4000 per year, between £4001- £8000 per year, more than £8000 per year)

Instruction A.3.10.2: single-choice question and only if A.3.10=A policy where the limit is *per condition*.

A.3.10.2: What is the insurance limit for your cat(s)? *If the limit is not the same for all cats, then think about the cat whose name comes first in the alphabet.*
Scroll-down menu (I don't know, up to £1500 per condition, between £1501- £4000 per condition, between £4001- £8000 per condition, more than £8000 per condition)

Instruction A.4: multiple choice question and only if A1=No
A.4: Why you do not have a dog and/or a cat?

I do not like dogs and/or cats.	
Someone I live with does not like dogs and/or cats.	
I do not have time to care for a dog and/or cat.	
I cannot afford to care for a dog and/or cat.	
I / someone in my household have/ has allergies that would be affected.	
I am not allowed to keep dogs and/or cats in my accommodation.	
I live in a flat and I do not think it is fair to keep a dog or a cat in that setting.	
I believe that people should not keep pets at all because it violates animals' rights.	
Other reasons	

Instruction A.4.1: open text field and only if A4="Other reasons"
You are welcome to explain why you don't have a dog or cat.

**Open response:**	
**I have no more to add.**	

Instruction A.5: drop-down menu from 18 - 100 years + "prefer not to say"
A.5: Please enter your current age in years from the drop-down menu.

_________ years old.

Instruction A.6: single-choice question

A.6: Please indicate your gender.

Male	
Female	
Neither of these	
Prefer not to say	

Instruction A.7: single-choice question

A.7: In which area is your primary place of residence?

For UK:

Scotland	
Northern Ireland	
Wales	
England - North East	
England - North West	
England - Yorkshire and Humberside	
England - West Midlands	
England - East Midlands	
England - South West	
England - South East	
England - Greater London	

Instruction A.8: single-choice question

A.8: Do you / did you work in the veterinary field (e.g. practicing veterinarian, veterinary nurse, veterinary assistant)?

Yes	
No	

Instruction A.10.1: single-choice question

A.10.1: Do you live alone (not including any pets)?

Yes	
No	

Instruction A.10.2: single-choice question and only if A10.1=No and list in drop-down-menu

A.10.2: Who do you live with?

I live with one or more adult(s)	
I live with one or more adult(s) and one child below 18 years (full time)	
I live with one or more adult(s) and one child below 18 years (part-time)	
I live with one or more adult(s) and children below 18 years (full time)	
I live with one or more adult(s) and children below 18 years (part-time)	
I live with one child below 18 years (full time)	
I live with one child below 18 years (part time)	
I live with children below 18 years (full time)	
I live with children below 18 years (part time)	
Other	

Instruction A11.1: single-choice question and only if A2=Dog & A10.2 = "I live with one or more adult(s)" or "I live with one or more adult(s) and one child below 18 years (full time)" or "I live with one or more adult(s) and one child below 18 years (part-time)" or "I live with one or

more adult(s) and children below 18 years (full time)" or "I live with one or more adult(s) and children below 18 years (part-time)" or "I live with one child below 18 years (full time)" or "I live with one child below 18 years (part time)" or "I live with children below 18 years (full time)" or "I live with children below 18 years (part time)"

A.11.1: For whom was/were the dog(s) acquired?

All (or most) in the household	
Me	
My partner/spouse	
My child / step child	
My children / step children	
For (an)other pet(s) in the household (as a companion)	
Others	
I don't know	

Instruction A11.2: single-choice question and only if A2=Cat & A10.2 = "I live with one or more adult(s)" or "I live with one or more adult(s) and one child below 18 years (full time)" or "I live with one or more adult(s) and one child below 18 years (part-time)" or "I live with one or more adult(s) and children below 18 years (full time)" or "I live with one or more adult(s) and children below 18 years (part-time)" or "I live with one child below 18 years (full time)" or "I live with one child below 18 years (part time)" or "I live with children below 18 years (full time)" or "I live with children below 18 years (part time)"

A.11.2: For whom was/were the cat(s) acquired?

All (or most) in the household	
Me	
My partner/spouse	
My child / step child	
My children / step children	
For (an)other pet(s) in the household (as a companion)	
Others	
I don't know	

Instruction A12.1: single-choice question and only if A2=Dog & A10.2 = "I live with one or more adult(s)" or "I live with one or more adult(s) and one child below 18 years (full time)" or "I live with one or more adult(s) and one child below 18 years (part-time)" or "I live with one or more adult(s) and children below 18 years (full time)" or "I live with one or more adult(s) and children below 18 years (part-time)" or "I live with one child below 18 years (full time)" or "I live with one child below 18 years (part time)" or "I live with children below 18 years (full time)" or "I live with children below 18 years (part time)"

A.12.1: Who in the household do you think is most attached to the dog(s)?

All (or most) in the household	
Me	
My partner/spouse	
My child / step child	
My children / step children	
Others	
I don't know	

Instruction A12.2: single-choice question and only if A2=Cat & A10.2 = "I live with one or more adult(s)" or "I live with one or more adult(s) and one child below 18 years (full time)" or "I live with one or more adult(s) and one child below 18 years (part-time)" or "I live with one or more adult(s) and children below 18 years (full time)" or "I live with one or more adult(s) and children below 18 years (part-time)" or "I live

with one child below 18 years (full time)" or "I live with one child below 18 years (part time)" or "I live with children below 18 years (full time)" or "I live with children below 18 years (part time)"

A.12.2: Who in the household do you think is most attached to the cat(s)?

All (or most) in the household	
Me	
My partner/spouse	
My child / step child	
My children / step children	
Others	
I don't know	

Instruction A13.1: multiple choice question and only if A2=Dog

A.13.1: Why was/were the dog(s) acquired? Choose all that are relevant for you.

To provide companionship (for humans)	
As company for other pet(s)	
To breed from	
To show in competitions / exhibitions	
For dog sports (training, agility, etc.)	
To exercise with (walks)	
As a hunting dog	
To protect home and/or property	
Other	
I don't know	

Instruction A13.2: multiple choice question and only if A2=Cat

A.13.2: Why was/were the cat(s) acquired? Choose all that are relevant for you.

To provide companionship (for humans)	
As company for other pet(s)	
To breed from	
To show in competitions / exhibitions	
To keep rodents away from the home	
It/they just appeared at my home and stayed	
Other	
I don't know	

Instruction 14.1: only if
Number of pet species=1 & A2Dog=1
Number of pet species=1 & A2Cat=1
Number of pet species=1 & A2Horse=1
Number of pet species=1 & A2Rabbit=1
Number of pet species=1 & A2Rodent=1
Number of pet species=1 & A2Bird=1
Number of pet species=1 & A2Reptile=1
Number of pet species=1 & A2Fish=1
Number of pet species=1 & A2Other pet than the above mentioned =1

A14.1: Please think about your pet and indicate whether you strongly disagree, somewhat disagree, somewhat agree, or strongly agree in the following statements.

Please use answer options /matrix provided under question A.14.11.1

Instruction 14.2: only if Number of pet species=1 & A2Dog>1
A14.2: Please think about your favourite dog and indicate whether you strongly disagree, somewhat disagree, somewhat agree, or strongly agree in the following statements.

Please use answer options /matrix provided under question A.14.11.1

Instruction 14.3: only if Number of pet species=1 & A2Cat>1
A14.3: Please think about your favourite cat and indicate whether you strongly disagree, somewhat disagree, somewhat agree, or strongly agree in the following statements.

Please use answer options /matrix provided under question A.14.11.1

Instruction 14.4: only if Number of pet species=1 & A2Horse>1
A14.4: Please think about your favourite horse and indicate whether you strongly disagree, somewhat disagree, somewhat agree, or strongly agree in the following statements.

Please use answer options /matrix provided under question A.14.11.1

Instruction 14.5: only if Number of pet species=1 & A2Rabbit>1
A14.5: Please think about your favourite rabbit and indicate whether you strongly disagree, somewhat disagree, somewhat agree, or strongly agree in the following statements.

Please use answer options /matrix provided under question A.14.11.1

Instruction 14.6: only if Number of pet species=1 & A2Rodent>1
A14.6: Please think about your favourite rodent and indicate whether you strongly disagree, somewhat disagree, somewhat agree, or strongly agree in the following statements.

Please use answer options /matrix provided under question A.14.11.1

Instruction 14.7: only if Number of pet species=1 & A2Bird>1
A14.7: Please think about your favourite bird and indicate whether you strongly disagree, somewhat disagree, somewhat agree, or strongly agree in the following statements.

Please use answer options /matrix provided under question A.14.11.1

Instruction 14.8: only if Number of pet species=1 & A2Reptile>1
A14.8: Please think about your favourite reptile and indicate whether you strongly disagree, somewhat disagree, somewhat agree, or strongly agree e in the following statements.

Please use answer options /matrix provided under question A.14.11.1

Instruction 14.9: only if Number of pet species=1 & A2Fish>1
A14.9: Please think about your favourite fish and indicate whether you strongly disagree, somewhat disagree, somewhat agree, or strongly agree in the following statements.

Please use answer options /matrix provided under question A.14.11.1

Instruction 14.10: only if Number of pet species=1 & A2Other pet than the above mentioned >1
A14.10: Please think about your favourite pet and indicate whether you strongly disagree, somewhat disagree, somewhat agree, or strongly agree in the following statements.

Please use answer options /matrix provided under question A.14.11.1

Instruction A.14.11: only if Number of pet species>1
A14.11: Now we would like you to think about your favourite pet. What species is your favourite pet?

Dog	
Cat	
Horse	

Rabbit	
Rodent (e.g. hamster, guinea pig, chinchilla, mouse/rat)	
Bird	
Reptile (e.g. lizard, snake, turtle)	
Fish (both aquarium fish and fish in garden pond)	
Other pet than the above mentioned	

Instruction 14.11.1: only if Number of pet species>1

A14.11.1: Now please indicate whether you strongly disagree, somewhat disagree, somewhat agree, or strongly agree to the following statements about your favourite pet.

1=strongly disagree, 2=somewhat disagree, 3=somewhat agree and 4=strongly agree

	**Attachment to my pet**	**1**	**2**	**3**	**4**
1	My pet means more to me than any of my friends				
2	Quite often I confide in my pet				
3	I believe that pets should have the same rights and privileges as family members				
4	I believe my pet is my best friend				
5	Quite often, my feelings towards people are affected by how they react to my pet				
6	I love my pet because he/she is more loyal to me than most of the people in my life				
7	I enjoy showing other people pictures of my pet				
8	I think my pet is just a pet				
9	I love my pet because it never judges me				
10	My pet knows when I'm feeling bad				
11	I often talk to other people about my pet				
12	My pet understands me				
13	I believe that loving my pet helps me stay heathy				
14	Pets deserve as much respect as humans do				
15	My pet and I have a very close relationship				
16	I would do almost anything to take care of my pet				
17	I play with my pet quite often				
18	I consider my pet to be great companion				
19	My pet makes me feel happy				
20	I feel that my pet is part of my family				
21	I am not very attached to my pet				
22	Owning a pet adds to my happiness				
23	I consider my pet to be a friend				

Instruction A15.1: only if A2=Dog

A.15.1: Please indicate which type of veterinary practice you usually attend with your dog(s):

Instruction A15.2: only if A2=Cat

A.15.2: Please indicate which type of veterinary practice you usually attend with your cat(s):

For UK:

Small animal practice (1-3 veterinarians)	
Small animal practice (4 or more veterinarians)	
Mixed practice (where other types of animals are also treated e.g. farm animals)	
University hospital	
Charity clinic	
I attend several types of practice.	
I do not have a veterinary practice that I attend	
I don't know	

Instruction A.16: only if A2=Dog &/or A2=Cat

A.16: How important are the following aspects to you when thinking about your veterinarian?

1= strongly disagree; 2 = disagree; 3 = somewhat disagree; 4 = neutral (neither agree nor disagree); 5 = somewhat agree; 6 = agree and 7 = strongly agree; 8=I don't know

	**It is important that…**	1	2	3	4	5	6	7	8
1	I see the same veterinarian every time.								
2	I have a good relationship with / like my veterinarian.								
3	my animal likes /seems comfortable with my veterinarian.								
4	my veterinarian seems to care about my pet								
5	my veterinarian has specialist qualifications in addition to their veterinary degree								
6	my vet has a professional manner								
7	I can trust my veterinarian								

Instruction A.17.: only if A2=Dog &/or A2=Cat

A.17: How important are the following aspects to you when choosing a practice?

1= "Not important at all"; 2="less important"; 3=" important"; 4=" really important"; 5=" I don't know"

	**The practice should…**	**1**	**2**	**3**	**4**	**5**
1	▪ be near where I live					
2	▪ provide 24 hour care for routine emergencies themselves (not divert me elsewhere)					
3	▪ be accessible with good parking					
4	▪ have appointments available at convenient times					
5	▪ consider species-related requirements (e.g. separate waiting room for cats to reduce stress)					
6	▪ offer specialist care if required e.g. joint replacements or cancer treatments					
7	▪ offer alternative medicine e.g. traditional Chinese medicine, homeopathy					
8	▪ charge reasonable prices					
9	▪ be independently-owned					
10	▪ be corporate-owned e.g. AniCura, IVC *(only UK IVC)*					
11	▪ make house calls					
12	▪ offer payment plans					
13	▪ make direct insurance claims					

Instruction A.18: multiple-choice question and only if A2=Dog &/or A2=Cat

A.18: Which of the following diagnostic and treatment options would you expect to be available in the practice you usually attend? Please tick all that apply.

**Radiography** (x-rays)	
**Ultrasound** (an imaging technique commonly used for examining the abdomen)	
**Endoscopy** (camera to look inside body, remove foreign bodies from passageways e.g stomach, airway - enables 'keyhole' surgery).	
**Arthroscopy** (camera to look inside joints)	
**In-house laboratory** (e.g. for blood tests, urine analysis)	
**Dental equipment**	
**MRI** scanner (an advanced imaging technique commonly used for the spine)	
**CT scanner** (an advanced an advanced imaging technique commonly used for the chest)	
**None of the above**	
**I don't know**	

Instruction A.19.1: single-choice question and only if A2=Dog

A.19.1: Have you ever taken your dog(s) to a specialist veterinarian (e.g. neurologist, behaviourist, oncologist)?

Yes	
No	
I don't know	

Instruction A.19.2: single-choice question and only if A2=Cat

A.19.2: Have you ever taken your cat(s) to a specialist veterinarian (e.g. neurologist, behaviourist, oncologist)?

Yes	
No	
I don't know	

Instruction A.20.1: single-choice question and only if A2=Dog & A.3.1-A.3.4Health Insurance=No or A.3.1-A3.4 Health Insurance=Not anymore

A.20.1: If (one of) your uninsured dog(s) was suffering from a severe illness, and would have to either undergo treatment (with a good chance of a successful outcome), or be euthanised, what would you do?

I would ask for euthanasia.	
I would spend < £100 on treatment.	
I would spend £101-500 on treatment.	
I would spend £501-1000 on treatment.	
I would spend £1001-3000 on treatment.	
I would spend £3001-5000 on treatment.	
I would spend £5001-79999 on treatment.	
I would spend £8000 or more on treatment.	
I don't know	

Instruction A.20.2: single-choice question and only if A2=Cat & A.3.6-A.3.9Health Insurance=No or A.3.6-A3.9Health Insurance=Not anymore

A.20.2: If (one of) your uninsured cat(s) was suffering from a severe illness, and would have to either undergo treatment (with a good chance of a successful outcome), or be euthanised, what would you do?

UK:

I would ask for euthanasia.	
I would spend < £100 on treatment.	
I would spend £101-500 on treatment.	
I would spend £501-1000 on treatment.	
I would spend £1001-3000 on treatment.	
I would spend £3001-5000 on treatment.	
I would spend £5001-7999 on treatment.	
I would spend £8000 or more on treatment.	
I don't know	

Instruction A.21.1: single-choice question and only if A2=Dog & A.3.1-A.3.4Health Insurance=Yes

A.21.1: If (one of) your insured dog(s) were suffering from a severe illness, and would have to either undergo treatment (with a good chance of a successful outcome), or be euthanised, what would you do?

Instruction A.21.2: single-choice question and only if A2=Cat & A.3.6-A.3.9Health Insurance=Yes

A.21.2: If (one of) your insured cat(s) were suffering from a severe illness, and would have to either undergo treatment (with a good chance of a successful outcome), or be euthanised, what would you do?

UK:

I would ask for euthanasia.	

**I would spend < £100 above the insurance maximum.**	
**I would spend £101-500 above the insurance maximum.**	
**I would spend £501-1000 above the insurance maximum.**	
**I would spend £1001-3000 above the insurance maximum.**	
**I would spend £3001-5000 above the insurance maximum.**	
**I would spend £5001-7999 above the insurance maximum.**	
**I would spend £8000 or more above the insurance maximum.**	
**I don't know**	

Instruction Section B: only if A2=Dog & A2=Cat

SECTION B:

This second part of the questionnaire focuses on the advanced veterinary treatments available in modern veterinary practice.

B.1: Advances in small animal practice

B.1: To what extent do you agree with the following statements:

1= strongly disagree; 2 = disagree; 3 = somewhat disagree; 4 = neutral (neither agree nor disagree); 5 = somewhat agree; 6 = agree and 7 = strongly agree

		1	2	3	4	5	6	7
1	My pet should have access to the same **diagnostic tests** that are available to human patients							
2	My pet should have access to the same **treatment options** that are available to human patients							
3	I would enrol my pet in a research study to help advance veterinary care, as long as the risk of potential complications was low.							
4	My vet should offer my pet the most advanced treatment that is available.							
5	It is important that my vet contributes knowledge to the advancement of veterinary care for future patients.							
6	The advanced care available in modern veterinary medicine has gone 'too far', putting animals through 'too much'							
7	The advanced care available in modern veterinary medicine is unnecessary - animals should not be treated in the same way as humans.							

B.2: Factors related to the patient, the client and veterinarian's professional environment

B.2: In respect of your veterinarian's approach, to what extent do you agree with the following statements:

1= strongly disagree; 2 = disagree; 3 = somewhat disagree; 4 = neutral (neither agree nor disagree); 5 = somewhat agree; 6 = agree and 7 = strongly agree

		1	2	3	4	5	6	7
1	My pet should always be the priority for my vet when making medical decisions.							
2	If my pet was terminally ill, my vet should be willing to provide palliative care to allow me time to say 'goodbye'.							
3	My vet should discuss all appropriate treatment options with me, but it is up to me to decide.							
4	My vet should consider my emotional concerns during the decision-making process.							
5	My vet should encourage me to provide the best care for my pet, even if it might put me under pressure e.g. time-related or financially.							
6	My vet should consider my personal situation / concerns in the decision-making process, even if this means not being able to deliver the best possible treatment for my pet.							
7	My vet should only make decisions that are in the best interests of my pet, even if this means sending me to another vet.							
8	It is my vet's responsibility to find the best possible solution for my pet **and** me.							
9	My vet should not only act professionally but should also be emotionally supportive of me.							
10	If my vet's opinion differs from mine, they should ultimately let me decide.							
11	It is more important that my vet acts in the best interest of my pet, than considers my concerns (e.g. financial issues).							
12	In case of conflict, my vet should empathise with my personal feelings during the decision-making process.							
13	My vet should not make the decisions - I should make the decisions for my pet							
14	My vet should empathise with my situation in the decision-making process.							
15	My vet should be willing to make compromises in patient care if I cannot afford the best possible/ most appropriate treatment.							

B.2.1: Regarding veterinary advice

Instruction B.2.1: single-choice question

B.2.1: Have you ever been in a situation where you wanted to continue with treatment for your pet against your vet's advice?

Yes	
No	
I don't know	

Instruction B.2.2: only if B.2.1=Yes

B.2.2: To what extent did the following factors influence your desire to continue treatment against your vet's advice?

1= not at all; 2 = slightly; 3 = moderately; 4 = relatively strongly; 5 = very strongly and 6 = I don't know

		1	2	3	4	5	6
1	I know my pet better than my vet does						
2	Pressure from another person (e.g. family member, friend)						
3	I obtained medical information from the internet						
4	I believe(d) it to be in the best interests of my animal						

5	I have had / heard of a positive experience with another animal having the same treatment						
6	I have had / heard of a positive experience with family or friends going through the same treatment						
7	I have seen positive reports about the treatment in the press and social media						
8	I obtained a second opinion from another veterinarian						
9	My animal was insured						

Instruction B.2.3: single-choice question

B.2.3: Have you ever been in a situation where you refused treatment for your pet that your vet recommended?

Yes	
No	
I don't know	

Instruction B.2.4: only if B.2.3=Yes

B.2.4: To what extent did the following factors influence your decision to refuse treatment that your vet recommended?

1 = not at all; 2 = slightly; 3 = moderately; 4 = relatively strongly; 5 = very strongly and 6 = I don't know

		1	2	3	4	5	6
1	I could not afford the treatment at the time						
2	I felt that my animal was too old						
3	I was not strongly emotionally attached to my animal						
4	Pressure from another person (e.g. family member, friend)						
5	I have / had limited time resources to provide the required care						

6	I did not think that the treatment was in my animal's best interests						
7	I have had / heard of a bad experience with another animal going through the same treatment						
8	I have had / heard of a bad experience with family or friends going through the same treatment						
9	I have seen negative reports about the treatment in the press and social media						
10	I wanted to obtain a second opinion from a different veterinarian						

B.3: Health insurance in veterinary practice

Instruction B.3.1: multiple choice question and only if A3.1= Health insurance=yes or A.3.2.1Health insurance=Yes or A.3.2.2Health insurance=Yes or A.3.3.1Health insurance=Yes or A3.3.2Health insurance=Yes or A3.3.3Health insurance=Yes or A.3.4.1Health insurance=Yes or A3.4.2Health insurance=Yes or A3.4.3Health insurance=Yes or A.3.6=Health insurance=Yes or A.3.7.1Health insurance=Yes or A.3.7.2Health insurance=Yes or A.3.8.1Health insurance=Yes or A3.8.2Health insurance=Yes or A3.8.3Health insurance=Yes or A.3.9.1Health insurance=Yes or A3.9.2Health insurance=Yes or A3.9.3Health insurance=Yes

B.3.1 Why do you have a health insurance for your pet(s)? Please tick all that apply.

1	My vet advised me to take out health insurance	
2	I have had insurance for pets previously	
3	I have been in the situation of not being able to afford treatment before	
4	Friends advised me to take out health insurance	
5	I believe it is part of being a responsible owner	
6	I may not be able to afford veterinary care for my pet without it	
7	Because my pet is old and at greater risk of illness	
8	To avoid having to make difficult financial decisions if my pet becomes ill	

9	Insurance was included free /very cheap when I first obtained my pet (e.g. puppy package)	
10	I don't know	
11	Other	

Instruction B.3.2: multiple choice question and only if A3.1Health insurance=No or A.3.2.1Health insurance=No or A.3.2.2Health insurance=No or A.3.3.1=Health insurance=No or A3.3.2Health insurance=No or A3.3.3Health insurance=No or A.3.4.1Health insurance=No or A3.4.2Health insurance=No or A3.4.3Health insurance=No or A.3.6Health insurance=No or A.3.7.1Health insurance=No or A.3.7.2Health insurance=No or A.3.8.1Health insurance=No or A3.8.2Health insurance=No or A.3.8.3Health insurance=No or A.3.9.1Health insurance=No or A3.9.2Health insurance=No or A3.9.3Health insurance=No

B.3.2 Why do you not have health insurance for your pet(s)? Please tick all that apply.

1	I had not heard about it	
2	I do not trust insurance policies	
3	Pet insurance policies are too expensive	
4	My vet advised me not to purchase health insurance	
5	The benefits are not worth the cost	
6	It was too difficult to find a suitable policy	
7	My pet is too old, and so it is not relevant anymore	
8	My pet is too old, and insurance has become too expensive	
9	I don't know	
10	Other	

Instruction B.3.3: multiple choice question and only if A3.1Health insurance=not anymore A.3.2.1Health insurance=Not anymore or A.3.2.2Health insurance=Not anymore or A.3.3.1=Health insurance=Not anymore or A3.3.2Health insurance=Not anymore or A3.3.3Health insurance=Not anymore or A.3.4.1Health insurance=Not anymore or A3.4.2Health insurance=Not anymore or A3.4.3Health insurance=Not anymore or A3.6Health insurance=not anymore or A.3.7.1Health insurance=Not anymore or A.3.7.2Health insurance=Not anymore or

A.3.8.1Health insurance=Not anymore or A3.8.2Health insurance=Not anymore or A3.8.3Health insurance=Not anymore or A.3.9.1Health insurance=Not anymore or A3.9.2Health insurance=Not anymore or A3.9.3Health insurance=Not anymore

B.3.3 Why did you not continue to insure your pet(s)? Please tick all that apply.

1	The benefits were not worth the expense	
2	It became too expensive	
3	Too many exclusions were added	
4	Not all costs were covered	
5	I don't know	
6	Other	

B.4.: The use of social media by clients and veterinary practices

Social media are forms of electronic communication (e.g. Facebook, Twitter, Instagram) through which users create online communities to share information, ideas, personal messages, and other content (such as videos, photos).

Instruction B.4.1: multiple choice question

B.4.1: What sort of social media do you use, either casually or as a registered user? Tick all that apply.

1	I do not use any social media	
2	Facebook	
3	Instagram	
4	Twitter	
5	YouTube	
6	TikTok	
7	Other	

Instruction B.4.2: single-choice choice question and only if B4.1=Facebook, B4.1=Instagram, B4.1=Twitter, B4.1=YouTube, B4.1=TikTok and/or B4.1=other

B.4.2: How often do you use social media?

Every day	
4-6 days per week	
1-3 days per week	
Less than one day per week	
Less frequently	
I don’t know	

Instruction B.4.3: single-choice choice question

B.4.3: Do you expect your veterinary practice to have an active presence on social media?

Yes	
No	
I don’t know	

B.4.4: To what extent do you agree with the following statements?

1= strongly disagree; 2 = disagree; 3 = somewhat disagree; 4 = neutral (neither agree nor disagree); 5 = somewhat agree; 6 = agree; 7 = strongly agree and 8 = I don’t know

	**I think that the use of social media by veterinary practices…**	1	2	3	4	5	6	7	8
1	gives people an insight into what happens in the practice.								
2	is not necessary.								
3	is a useful way for pet owners to find a (new) practice.								

4	enables owners to stay in touch with the veterinary practice and/or the veterinarian in an informal and easy way.								

Instruction B.4.5: single-choice choice question

B.4.5: Have you ever left (a) negative comment(s) / complaint(s) about your vet / practice on the internet (e.g. webpages or social media)?

Yes	
No	

Instruction B.4.6: multiple choice question and only if B.4.5=yes

B.4.6: If yes, what was/were the negative comment(s) / complaint(s) about? Please tick all that apply.)

too long a waiting time	
the veterinarian’s medical advice	
the way my pet was handled by the staff	
the outcome of my pet’s treatment / complications	
the cost of treatment	
behaviour of specific staff member(s)	
the lack of technical equipment in the practice	
unfriendly communication by the veterinarian	
too technical or complicated way of communicating by the veterinarian	
unfriendly communication by other staff	
Other	

Instruction B.4.7: single-choice choice question

B.4.7: Did your vet respond to the negative feedback / complaint?

Yes	
No	
I don't know	

B.5: Using internet resources to find veterinary medical information

Instruction B.5.1: single-choice choice question

B.5.1: How often do you use internet resources to find medical information PRIOR to a consultation with your vet?

Never	
Occasionally	
Frequently	
Always	
I don't know	

Instruction B.5.2: single-choice choice question

B.5.2: How often do you use internet resources to find medical information AFTER consultation with your vet?

Never	
Occasionally	
Frequently	
Always	
I don't know	

Instruction B.5.3: multiple choice question and only if B.5.1 or B.5.2=occasionally, B.5.1 or B.5.2=frequently, B.5.1 or B.5.2=always and/or B.5.1 or B.5.2=I don't know

B.5.3: Which resources have you used?

1	Blogs and chat rooms	
2	Websites providing veterinary medical information	
3	Social media (e.g. Facebook, Twitter)	
4	Practice website	
5	Veterinary association websites (e.g. BVA, BSAVA, RCVS)	
6	University website	
7	Other	

Instruction B.5.4: only if B.5.1 or B.5.2=occasionally, B.5.1 or B.5.2=frequently, B.5.1 or B.5.2=always and/or B.5.1 or B.5.2=I don't know

B.5.4: To what extent do you agree with the following statements?

1= strongly disagree; 2 = disagree; 3 = somewhat disagree; 4 = neutral (neither agree nor disagree); 5 = somewhat agree; 6 = agree; 7 = strongly agree and 8 = I don't know

	**The use of internet resources…**	1	2	3	4	5	6	7	8
1	increases my expectations of the standard of veterinary care available for my pet.								
2	enables me to have a more informed discussion with my vet.								
3	can lead to situations where I am better informed than my vet.								
4	enables me to challenge my vet to justify their recommendations.								
5	helps me to make the right decision for my animal.								
6	enables me to buy some medication(s) more cheaply (e.g. flea treatments, wormers).								
7	can give the wrong impression of standard veterinary medicine								

Instruction B.5.5: single-choice question and only if B.5.1 or B.5.2=occasionally, B.5.1 or B.5.2=frequently, B.5.1 or B.5.2=always and/or B.5.1 or B.5.2=I don't know

B.5.5: Have you ever disagreed with your vet's professional advice based on information you obtained from the internet?

Yes	
No	
I don't know	

B.6: Telemedicine in modern small animal practice

Telemedicine, defined as the practice of using electronic media to deliver advice and care 'at a distance', **has become an option in modern small animal practice.** This enables animal owners to have a full consultation with a vet - for which they are charged - without having to take their pet to the practice.

Instruction B.6.1: single-choice question

B.6.1: Have you ever used telemedicine to obtain veterinary advice about your pet(s)?

Yes	
No	
I didn't know it was an option	

Instruction B.6.2: single-choice question and only if B.6.1=No or B.6.1=I didn't know it was an option

B.6.2: Would you make use of telemedicine if your vet offered it instead of an in-person consultation?

Yes	
No	
I don't know	

B.6.3: To what extent do you agree with the following statements?

1= strongly disagree; 2 = disagree; 3 = somewhat disagree; 4 = neutral (neither agree nor disagree); 5 = somewhat agree; 6 = agree; 7 = strongly agree and 8 = I don't know

	**The use of telemedicine…**	1	2	3	4	5	6	7	8
1	could be helpful, as I find it difficult to travel to a vet.								
2	could save my pet from a stressful journey to the vet.								
3	could help me decide whether my pet needs to see a vet.								
4	could be useful for follow-up appointment.								
5	could improve my access to a specialist if there are none locally.								
6	is good in case of emergencies.								
7	weakens the veterinarian-client bond.								
8	enhances the veterinarian-client bond.								
9	should cost less than a normal consultation.								
10	has no benefit.								
11	increases the risk of something being missed because the animal is not physically examined by the vet.								
12	is more convenient than attending in person.								
13	is not an option for me as I don't have the necessary technical IT skills or equipment.								

C: Social Support

Finally, we kindly ask you to provide some information concerning your social environment and support network.

C.1: If you needed it, how often is someone available…

1=never, 2=occasionally, 3= mostly and 4= always

		**1**	**2**	**3**	**4**
1	to take you to the doctor if you need it.				

2	to prepare your meals if you are unable to do it yourself.				
3	to help with daily chores if you were sick.				
4	to give you good advice about a crisis.				
5	to confide in or talk to about yourself or your problems.				
6	who understands your problems.				

Instruction C.2: single-choice question and list in drop-down menu

C.2: How often do you feel that you have no one to talk to?

Hardly ever or never	
Some of the time	
Often	

Instruction C.3: single-choice question and list in drop-down menu

C.3: How often do you feel left out?

Hardly ever or never	
Some of the time	
Often	

Instruction C.4: single-choice question and list in drop-down menu

C.4: How often do you feel isolated from others?

Hardly ever or never	
Some of the time	
Often	

Instruction C.5: single-choice question and list in drop-down menu

C.5: How often do you feel lonely?

Often/always	
Some of the time	
Occasionally	
Hardly ever	
Never	

You have now completed the actual questionnaire.
If you would like to add any further comments to the subject, please provide them below.

**Open response field:**	
**I have no further remarks.**	

Thank you for participating in the survey!
